# Supplementary material for: Multi-platform profiling characterizes molecular subgroups and resistance networks in chronic lymphocytic leukemia
Source: Nat Commun. 2021 Sep 13;12:5395. doi: 10.1038/s41467-021-25403-y (PMC8438057; doi:10.1038/s41467-021-25403-y)
Supplement: Supplementary file 1 — Supplementary Information [file 41467_2021_25403_MOESM1_ESM.pdf]

Supplementary Information

Supplementary Figures

Supplementary Figure 1a-d: Consensus clustering identifies dominant clusters in CLL
Supplementary Figure 1e: Analytic steps used for differentiation and validation of CLL subtypes
Supplementary Figure 1f: Genes defining CC based subtypes
Supplementary Figure 1g: DNA-based vs. mRNA-based class discovery
Supplementary Figure 2: Expression of *NRIP1* and genes with recurrent mutations, target gene prediction by GISTIC analysis for 11q deletions
Supplementary Figure 3: Specific analysis for processes involving cell cycle checkpoints, PI3K/RAS signaling, AID/APOBEC family members, tumor suppressor genes on 1p36
Supplementary Figure 4: Overexpression of AID induces genomic instability
Supplementary Figure 5: EMT-like regulatory network, transcriptional changes in tri(12) CLL, epigenetic modifiers in CLL subtypes
Supplementary Figure 6: Assessment of epigenetic regulation using GEP and DNA methylation analysis
Supplementary Figure 7: Clinical impact and differential response to treatment in CLL subtypes, CLL8 cohort
Supplementary Figure 8: PFS and OS by IGHV mutation status in CLL subtypes
Supplementary Figure 9: PFS by (I)EMT-L(C1), GI(C2) and cytogenetics (hierarchical model)
Supplementary Figure 10: PFS for GI and (I)EMT-L (*TP53* wild-type) and all cases with *TP53* mutation/deletion
Supplementary Figure 11: PFS for GI and (I)EMT-L (*TP53/ATM* wild-type), *TP53* and *ATM* mutation/deletion
Supplementary Figure 12: PFS for GI and (I)EMT-L *TP53* wild-type cases by mutation status of *SF3B1*
Supplementary Figure 13: Characteristic GEP of CLL subtypes are validated in the REACH cohort
Supplementary Figure 14: Clinical impact of CLL subtypes in relapsed cases, REACH cohort

Supplementary Figure 1: Consensus clustering identifies dominant clusters in CLL.

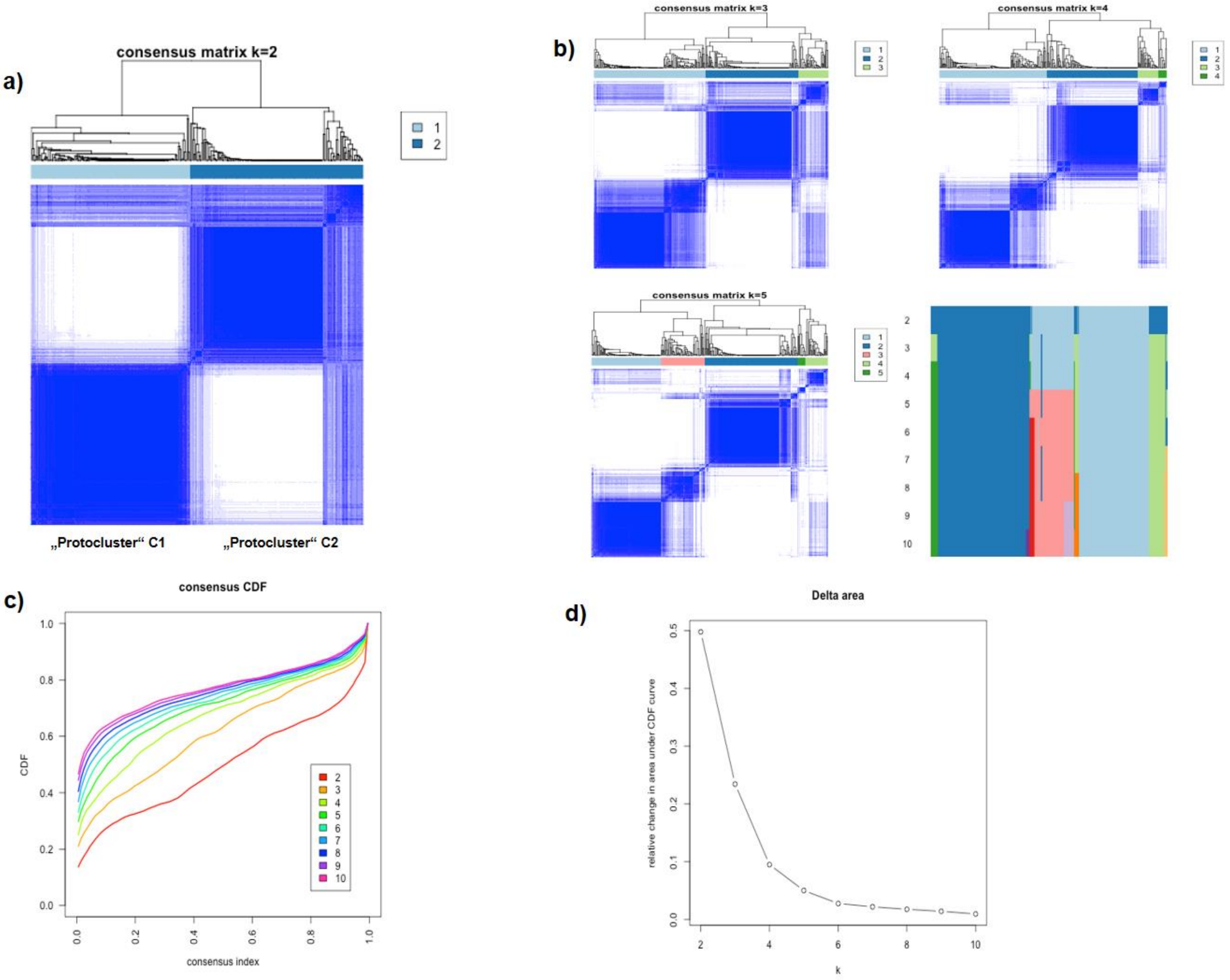

Supplementary Figure 1: Consensus clustering identifies dominant clusters in CLL.
a) Heatmap showing composition of gene expression profiles for two initial clusters (protoclusters) after consensus clustering (k=2) on 2359 transcripts. **b)** Consensus heatmaps for k=3, k=4, k=5 clusters showing cluster evolution and cluster relationship. Tracking plot (for up to k=10 clusters) indicates cluster assignment of cases (columns) for each k (rows), colors used for the consensus matrix class assignments are retained. Figure visualizes cluster evolution relative to protoclusters and subsequently evolving clusters, unstable membership of cases in clusters is indicated by frequent change of the class assignment. Cluster hierarchy provides information on biological differentiation. Exemplarily, C4(EMT-L) originates first from C2 protocluster, C5(EBF1-r) originates from C4(EMT-L), early emergence of the clusters and subsequent stability implies highly unique characteristics, both C4(EMT-L) and C5(EBF1-r) have features of C2(GI) since evolving from the same C2 protocluster. C2(GI) and C1((I)EMT-L) show the highest homogeneity and stability as indicated from the tracking plot, both clusters represent the highest distinction within the whole dataset (class labels provided in brackets refer to the biologic classes subsequently introduced). **c)** Empirical cumulative distribution function (CDF) for k=2-10 clusters, plot visualizes the curve of a consensus matrix, sample pairs which show decreased co-clustering are represented in decreasing frequency in the lower left portion, sample pairs which frequently cluster together are shown with increasing frequency in the upper right portion (high consensus index values). **d)** Delta area plot for changes in area under the CDF curve visualizing the change for k=2-10 clusters. Size of area under the CDF curve provides information for stability (decreasing stability with decreasing area). Degree of change for area under the CDF curve can be interpreted as representation of biological information. Exemplary, no change (flat curve) for increasing k would not provide additional description of represented data. Data within individual figures derives from n=337 biologically independent samples.

Supplementary Figure 1e: Analytic steps used for differentiation and validation of CLL subtypes.

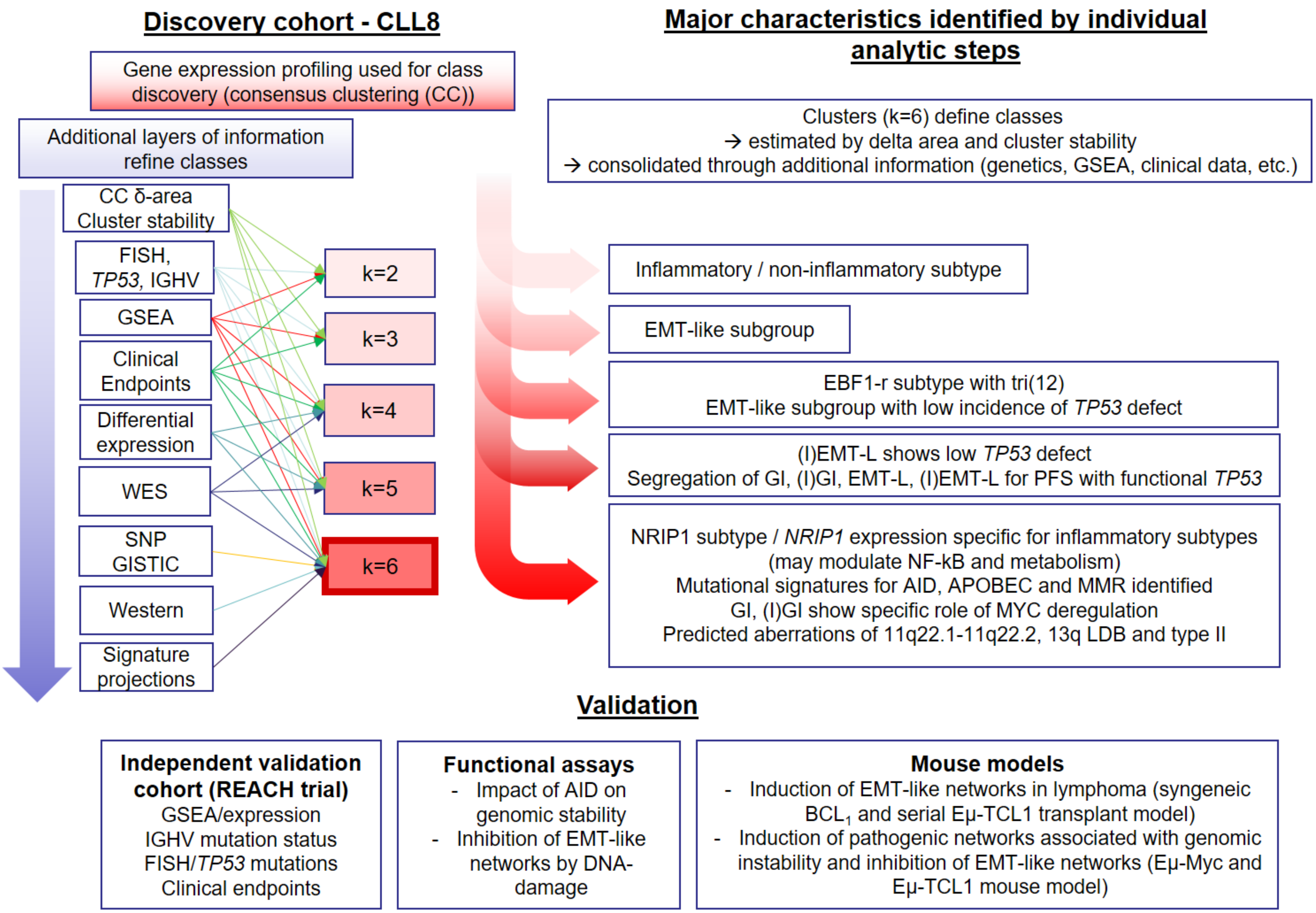

Supplementary Figure 1e: Analytic steps used for differentiation and validation of CLL subtypes.
The figure provides an extended overview of the strategy and successive workflow used for validation of gene expression based clusters and its detailed biologic characterization. Tumor heterogeneity was explored by performing consensus clustering (CLL8 GEP profiles). Initial estimate for optimal cluster numbers (k=6) was conducted by assessing delta-area and cluster stability. To assess the individual contribution of each cluster we conducted specific analyses for these clusters in a hierarchical fashion. Analyses for k=2-6 included the distribution of chromosomal aberrations assessed by FISH (identifies enrichment of del(17p) in C2(GI)/C3((I)GI), tri(12) in C5(EBF1-r)); for *TP53* and IGHV sequencing (identifies enrichment of *TP53* mutations in C2(GI)/C3((I)GI) and V3-21 usage in C3((I)GI)); GSEA for defining biologic categories (e.g. inflammatory/non-inflammatory subtype and genomically instable/EMT-like subgroup); and clinical endpoints (identifies differential response to treatment). Of note, C3((I)GI) segregated from C1 not before assessing k=5 clusters, however C3((I)GI) was showing considerable impact on the distribution of high-risk markers del(17p)/*TP53* mutation and refined segregation for clinical endpoints (therefore, subsequent analyses were only conducted for clusters emerging for k=4-6). Additional information was integrated for k=4-6 by assessing differentially expressed genes (identifying *EBF1, NRIP1* and other characteristic genes) and whole exome sequencing (WES) (e.g. identifying specific distribution of *POT1* mutations in C2(GI)/C3((I)GI). K=6 identified *NRIP1* as potential modulator of inflammation or energy consumption in the inflammatory or non-inflammatory subtype. For k=6 western blot was performed in select cases (validation of single genes like *PRMT5*, *XPO1*). Signature projections (e.g. AID and DNA damage associated signatures) and SNP/GISTIC analysis (enrichment for CNAs such as gains of 8q24.21 (*MYC*) in C2(GI)/C3((I)GI) or specific chromosome aberration pattern for del(13q) (showing enrichment of long distal breaks and type II deletions) was conducted only for classes resulting from k=6. Validation for k=6 clusters was conducted by using GEP of an independent trial cohort (REACH trial, n=300) and n=89 additional CLL8 patients. Functional and mouse models were used to validate the impact of AID on genomic stability, induction of EMT-like networks in lymphoma and inhibition of EMT-like networks through DNA-damage response. Results depicted throughout this work represent integrated data for k=6 in CLL8 and correspondingly in REACH.

Supplementary Figure 1f: Genes defining CC based subtypes.

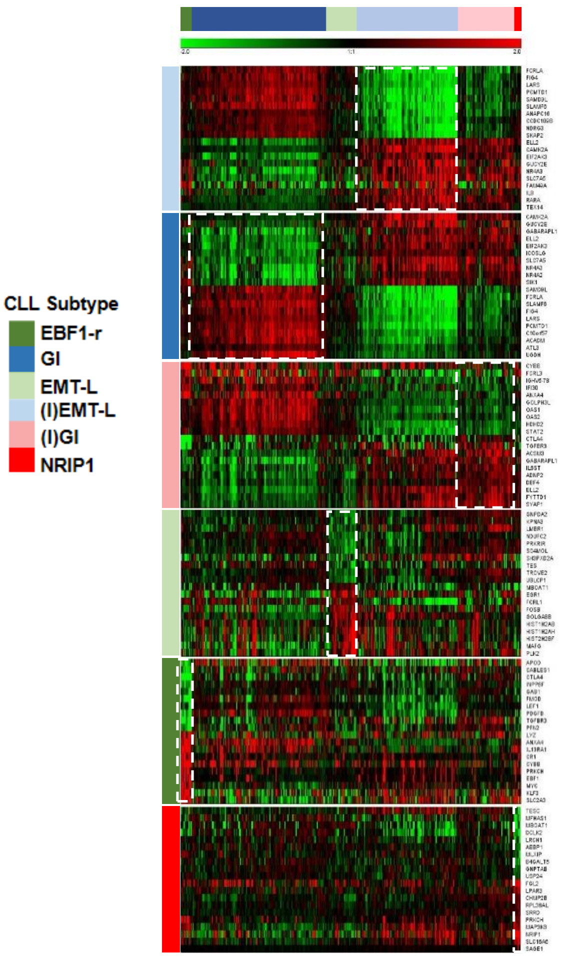

Supplementary Figure 1g: DNA-based vs. mRNA-based class discovery.

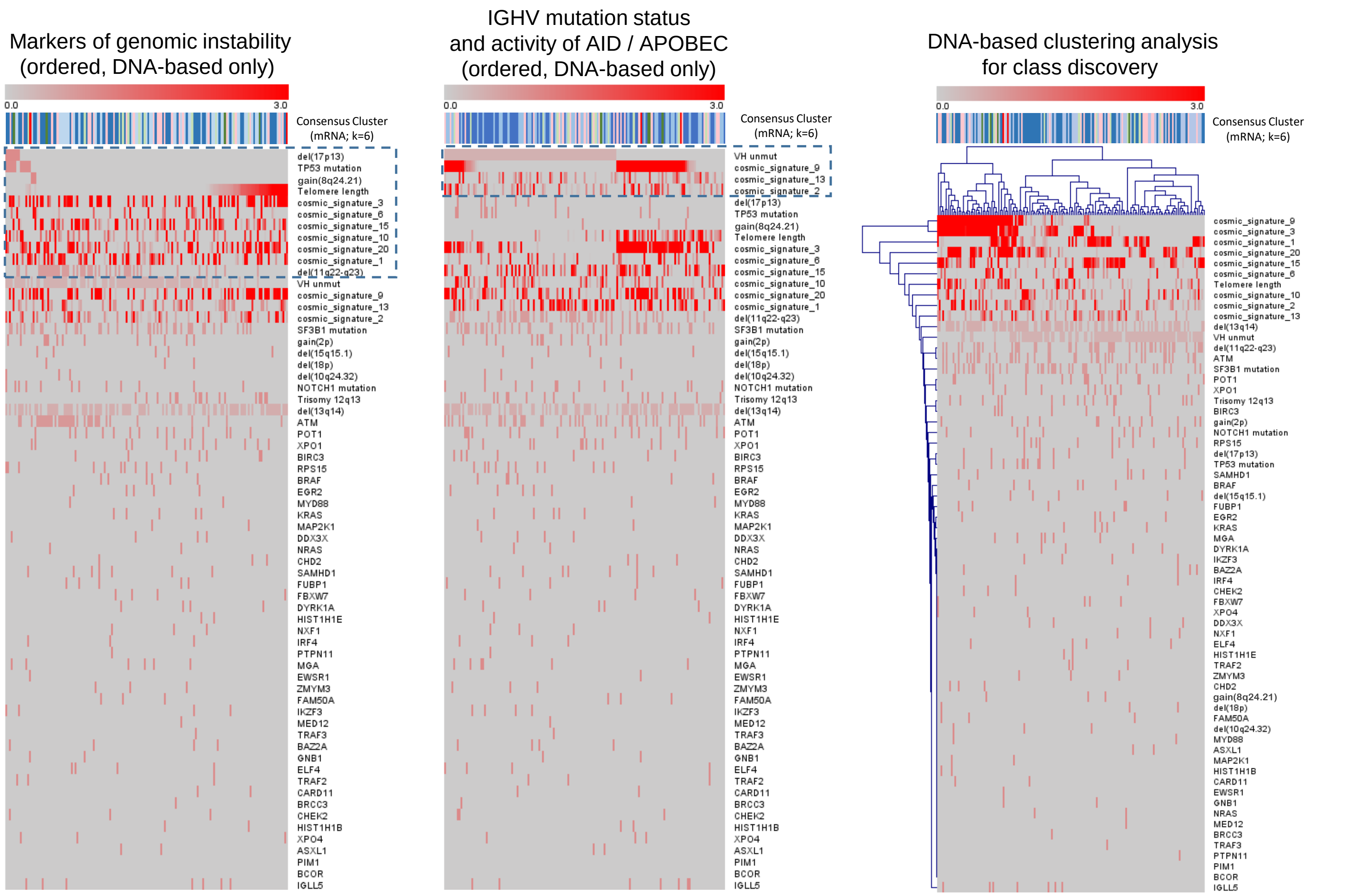

Supplementary Figure 1f: Genes defining CC based subtypes.
Heatmap showing top 10 up- and top 10 down-regulated genes found for individual clusters (calculated for individual cluster vs. remaining samples). Samples are ordered according to the consensus clustering (k=6) and indicated with the respective cluster color code (top row). Color coding on the left indicates the individual cluster gene set (top 10 up-/down-regulated genes). Single genes may overlap for different clusters. Data is shown for n=337 biologically independent samples.

Supplementary Figure 1g: DNA-based vs. mRNA-based class discovery.
DNA-based class discovery as independent approach shown in comparison to mRNA-based cluster identification. Depicted are three exploratory class discovery approaches using DNA-based parameters, such as IGHV and gene mutation status, chromosomal aberrations and mutational signature projections (n=162 cases with all parameters available). First panel depicts samples based on hierarchical order of parameters known to be associated with poor clinical course or genomic instability and signature projections inferring the activity of mutational processes which induce genomic instability (dotted box (top to bottom) highlights parameters used for ordering samples). Second panel depicts the hierarchical order of samples based on IGHV mutation status or mutational signature projections inferring the activity of AID and other APOBEC family members (dotted box (top to bottom) highlights parameters used for ordering samples). Third panel depicts the order of samples after clustering (hierarchical clustering with Euclidean distance and average linkage). In all panels top row indicates relative marker intensity range used to show individual DNA marker levels (for continuous variables based on mean intensity, color code used for binary variables indicates presence (red) or absence (grey)). Second row shows mRNA-based cluster assignment as found for consensus clustering with k=6. DNA-based parameters used for analysis are indicated on the right.

Supplementary Figure 2: Expression of *NRIP1* and genes with recurrent mutations, target gene prediction by GISTIC analysis for 11q deletions.

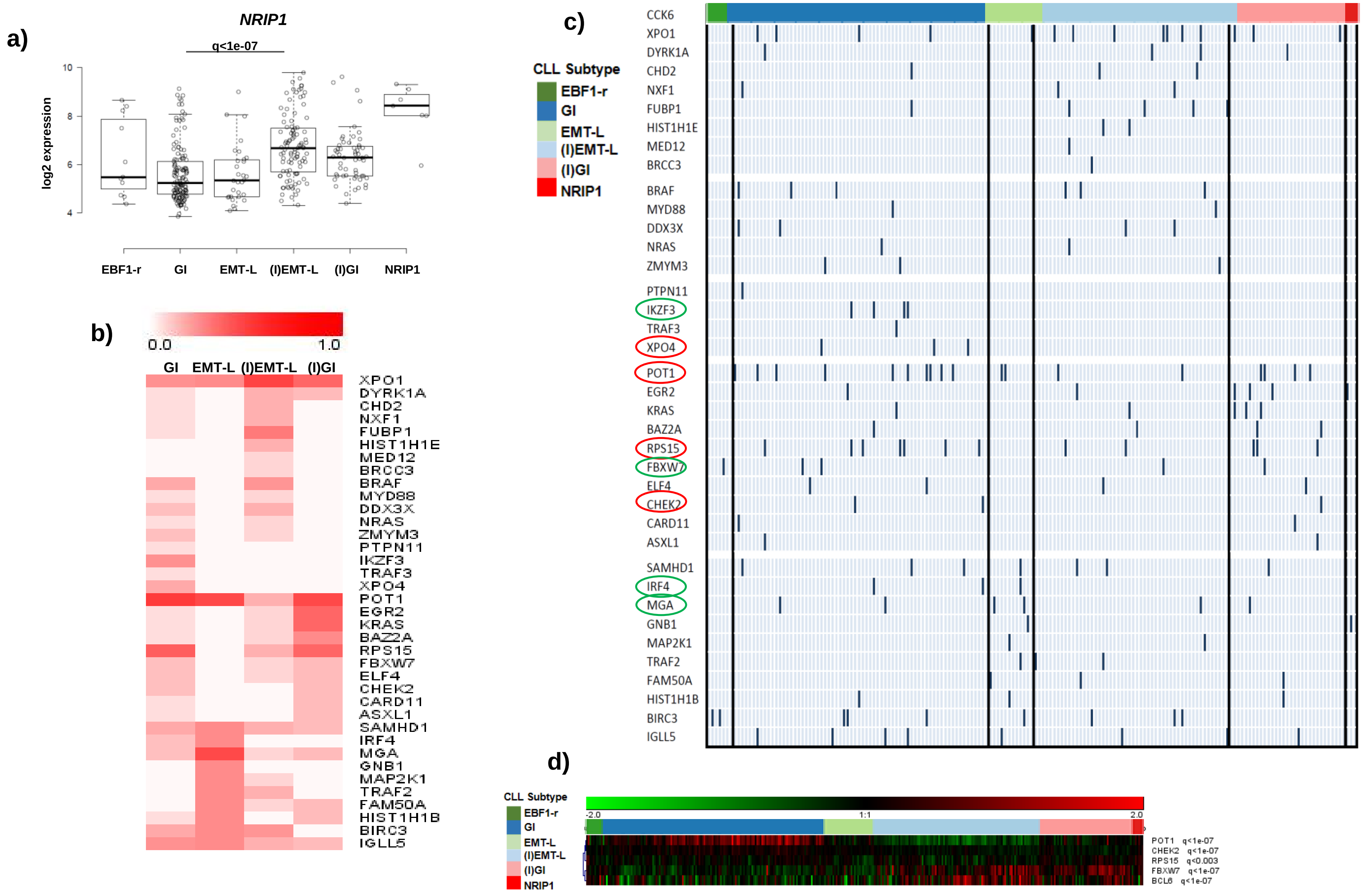

Supplementary Figure 2: Expression of *NRIP1* and genes with recurrent mutations, target gene prediction by GISTIC analysis for 11q deletions.
a) *NRIP1* expression with relation to major clusters. FDR for *NRIP1* from comparison of GI vs. (I)EMT-L is indicated (q<1e-07). Data is shown for n=337 biologically independent samples. For the boxplots, centerline, box limits, and whiskers represent the median, 25th, and 75th percentiles and 1.5x interquartile range, respectively. **b)** Figure displaying similarities (exclusivity and co-occurrence) for mutations regarding relative frequencies per cluster (higher frequency indicated with increasing color intensity) based on hierarchical agglomeration used for pattern discovery. Cluster with predominant enrichment of respective mutations is indicated in the header row. **c)** Detailed representation for mutations per case. Highlighted are mutations affecting genes involved in maintaining genomic stability (red circle) or involved in the regulation of MYC (green circle). **d)** Heatmap of selected genes with recurrent mutations in CLL show a cluster specific expression according to the underlying biology. FDRs of differentially expressed genes for GI vs. (I)EMT-L are indicated on the right (q).

f)

GI	(I)EMT-L; EMT-L; GI	EMT-L; GI	(I)GI; EMT-L; GI
MMP8	hsa-mir-34c	CWF19L2	C11orf65
TMEM133	FLJ46266	AASDHPPT	ACAT1
RBM7	POU2AF1	SLC35F2	NPAT
TRPC6	ARHGAP20	KBTBD3	ATM
ANKK1	ALG9	GRIA4	
LOC644672	C11orf1	CASP5	
CNTN5	CRYAB	CASP1	
HTR3B	PPP2R1B	CUL5	
KIAA1377	TIMM8B	PTS	
C11orf71	DLAT	ICEBERG	
TMEM123	FDX1	INCA	
NNMT	RDX	DDI1	
C11orf70	SDHD	GUCY1A2	
ANGPTL5	BCDO2	ELMOD1	
MMP1	HSPB2	SLN	
MMP7	DIXDC1	CASP4	
DCUN1D5	SNF1LK2	LOC643923	
MMP13	LOC399947	COP1	
YAP1	IL18	RAB39	
MMP12	LAYN	ALKBH8	
FAM55A	DDX10	KIAA1826	
MMP27	LOC91893		
TMPRSS5	PIH1D2		
PDGFD	C11orf57		
MMP3	ZC3H12C		
ZW10	FLJ45803		
REXO2	BTG4		
FAM55D	TEX12		
MMP20	C11orf52		
PGR	C11orf53		
DRD2			
USP28			
BIRC2			
MMP10			
TTC12			
ZBTB16			
HTR3A			
BIRC3			
DYNC2H1			
NCAM1			

e)

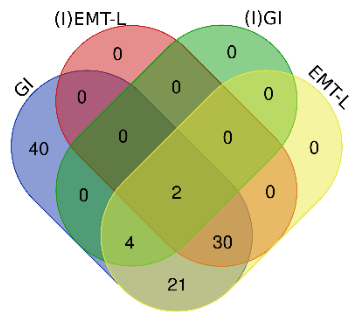

g)

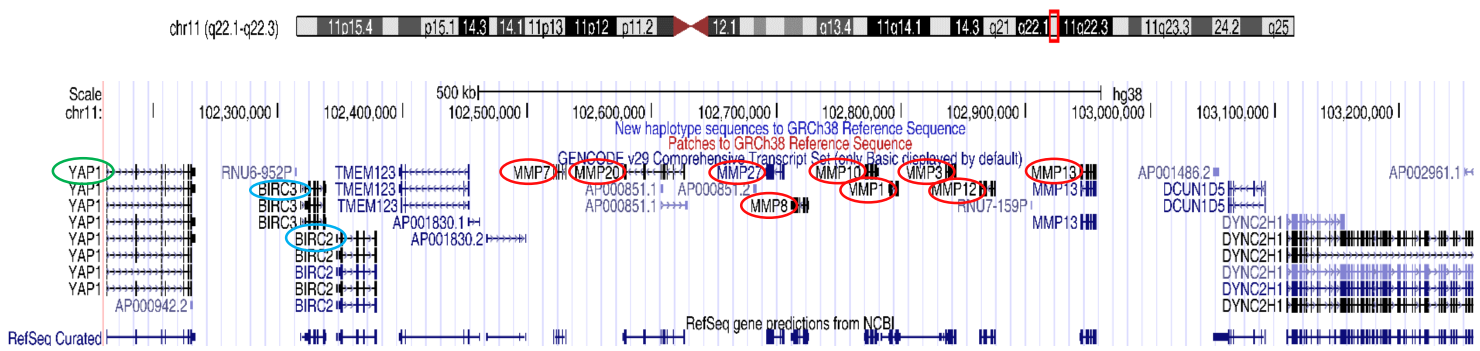

h)

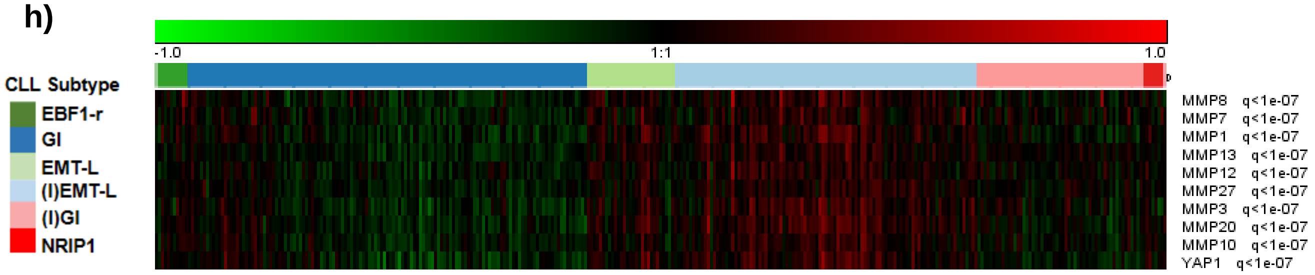

Supplementary Figure 2:

e) Venn diagram showing overlaps of candidate target genes (in wide peak region) identified for GI, EMT-L, (I)EMT-L and (I)GI for 11q deletions by GISTIC analysis of copy-number alterations. 40 genes are predicted as target genes exclusively affected in GI cases. **f)** List of target genes for 11q deletions and respective distribution as shown in the Venn diagram (Supplementary Fig. 2e) across classes, exclusive genes in GI involve a *MMP* cluster (red circles), *YAP1* (green circle), *BIRC2* and *BIRC3* (blue circles). **g)** Size position and location of genes were visualized by using the UCSC Genome Browser. Schematic representation of chromosome 11 and position of genes on 11q22.1-11q22.2 (red box). *MMPs* (red circles) are involved in e.g. extracellular matrix degradation during EMT, *YAP1* (green circle) is a transcriptional regulator for genes such as *BIRC2* or the EMT transcription factor *ZEB1*, *BIRC2* and *BIRC3* (blue circles) may be involved in activation of NF-kB signaling. **h)** Heatmap showing expression of *YAP1* and *MMPs*. In line with the target gene prediction by GISTIC, *YAP1* and *MMPs* are underexpressed in GI while EMT-L and (I)EMT-L show overexpression.

Supplementary Figure 3: Specific analysis for processes involved in genomic instability including cell cycle checkpoints, PI3K/RAS signaling, AID and APOBECs or tumour suppressor genes on 1p36.

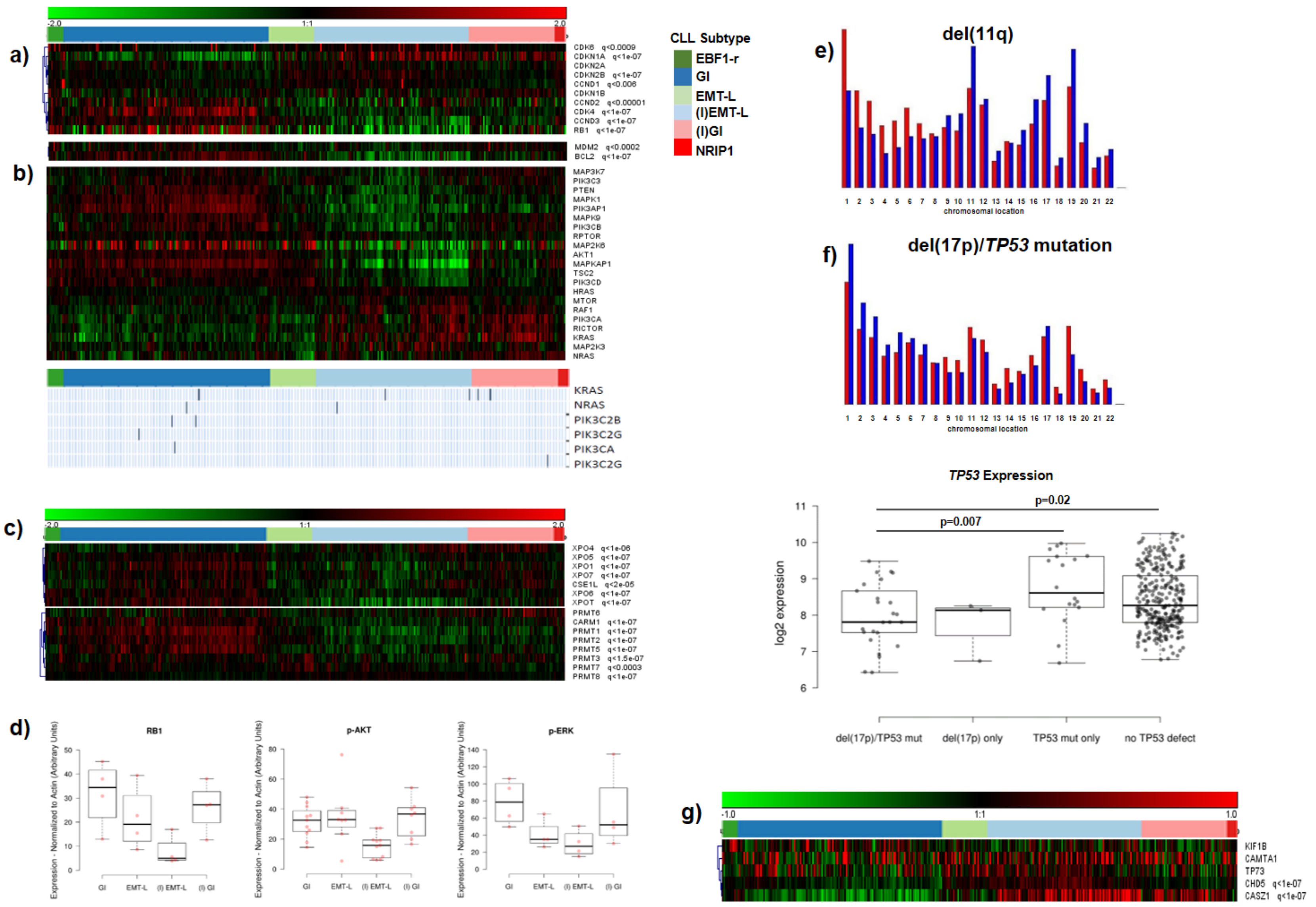

Supplementary Figure 3: a) Heatmap showing expression of cell cycle genes. Loss of *CDKN1A*, *CDKN2B* and overexpression of *CDK4*, *CCND2*, *CCND3*. Overexpression of *RB1* supports DNA damage associated induction. *RB1* inactivation can occur via phosphorylation by CDK4, methylation via SMYD2. Loss of *RB1* can be induced through type II or biallelic deletion. Overexpression of genes like *MDM2* and *BCL2* can induce or aggravate a dysregulated cell cycle by inhibition of p53 or apoptosis. CLL subtype color code defined in Supplementary Fig. 3a applies for Supplementary Fig. 3a-c/g. **b)** Expression profiles of genes contained in the GSEA gene sets associated with PI3K-AKT and RAS-ERK signaling. Mutations affecting *PIK3* family members are shown below expression profiles. These affect mostly class II kinases and show an enrichment in GI. *KRAS* mutations are preferentially found in cases, which cluster with genomic instable cases. **c)** Heatmap representing expression of exportin gene family members (*XPOs*) and protein arginine methyltransferases (*PRMTs*). FDRs of DEGs (GI vs. (I)EMT-L) are indicated on the right (q). **d)** Protein expression in CLL subtypes; RB1 (n=4 each), pERK (n=4 each), p-AKT (n=11 for GI/(I)EMT-L each, n=8 for (I)GI/EMT-L each) (normalized to actin). Shown are biologically independent samples without alterations besides del(13q). RB1 upregulation is found in GI cases without aberrations of the RB1 locus or other recurrent alterations and indicates a physiologic response to DNA damage. For the boxplots, centerline, box limits, and whiskers represent the median, 25th, and 75th percentiles and 1.5x interquartile range, respectively. **e/f**) Barplots showing differentially expressed genes (FDR<0.05) and the respective chromosomal distribution in cases with del(11q) and *TP53* defect (blue) in comparison to the complete (background filtered) dataset of expressed genes (red). Barplots show selective enrichment of genes positioned on chromosomes 19, 17 and 11 for del(11q) cases and on chromosomes 1, 2 and 3 for cases with *TP53* defect. Transcripts deregulated on chromosome 1 were frequently mapped on cytoband 1p36. *TP53* expression with regard to *TP53* mutation / deletion status: Cases with *TP53* deletion and mutation show lower expression in comparison to cases with sole *TP53* mutation (p=0.007, Mann-Whitney (two-sided)) or without alterations of *TP53* (p=0.02, Mann-Whitney (two-sided)). Shown are n=331 biologically independent samples. Boxplot/whiskers as defined in Supplementary Fig. 3d. **g)** Heatmap showing expression of putative tumor suppressor genes residing in chromosomal region 1p36, significant FDRs (q<1e-07) for downregulation (GI vs. (I)EMT-L) were found for *CASZ1* and *CHD5*.

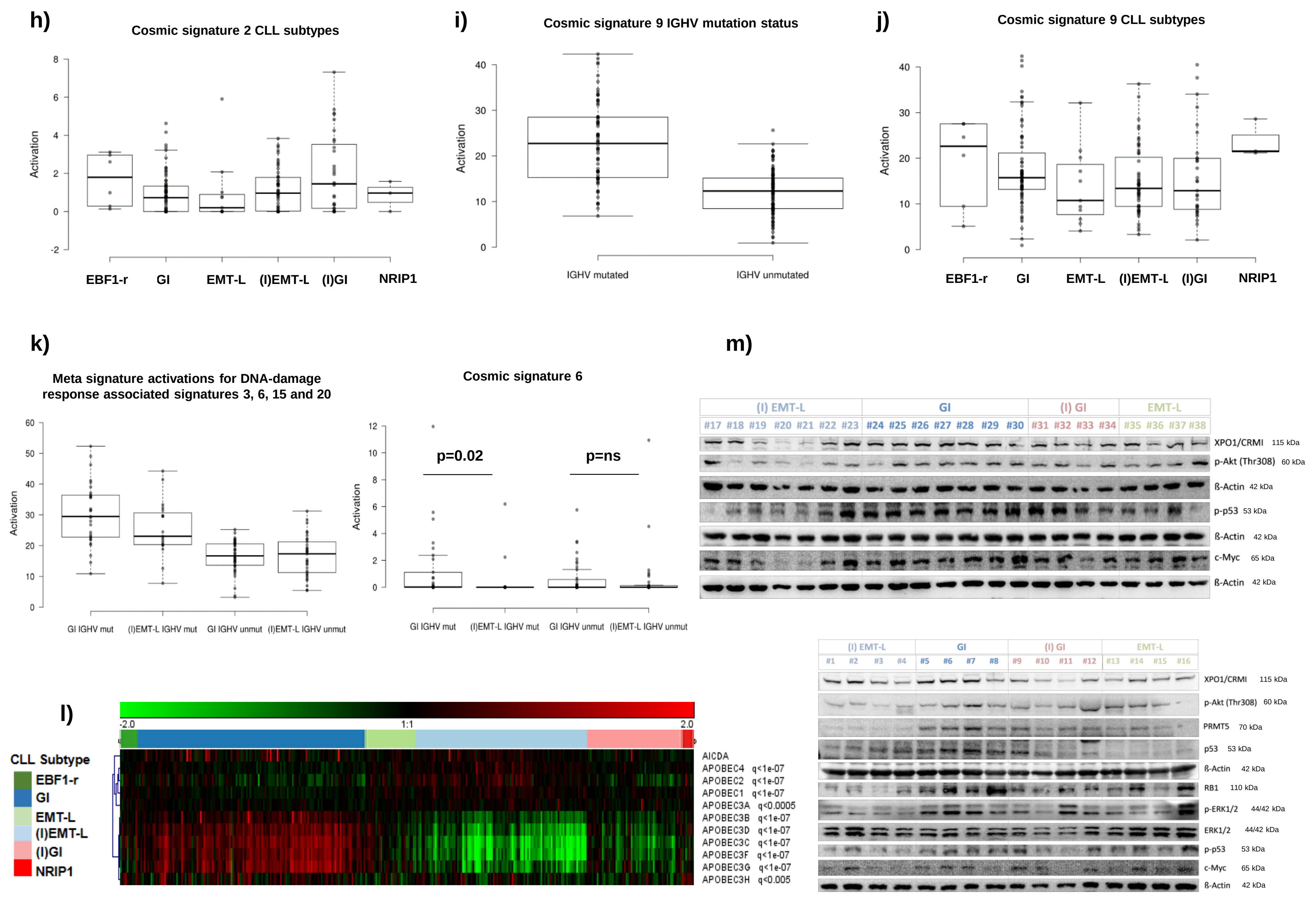

Supplementary Figure 3:

h/i/j) Activations of AID and APOBEC associated signatures 2 and 9 with regard to individual clusters or IGHV mutation status. **k)** Meta signature activations for DNA-damage response associated signatures 3, 6, 15 and 20 according to major clusters and IGHV status. IGHV mutated GI cases have higher overall activation compared to IGHV mutated (I)EMT-L cases with strong activation found for signature 6 (p=0.02, Mann-Whitney (two-sided)), while there was no difference for IGHV unmutated cases (ns: indicates not significant). For Supplementary Fig. 3h-k: data is shown for n=171 biologically independent samples; for the boxplots, centerline, box limits, and whiskers represent the median, 25th, and 75th percentiles and 1.5x interquartile range, respectively. **l)** Heatmap representing expression of *APOBEC* family members. Expression is not correlated with the (I)GI specific signature 2, in contrast to other cancers[1]. Induction of APOBEC family members is regulated through interferone in response to cytosolic (viral) DNA and signaling through the cGAS-STING pathway during the anti-viral response[2] and DNA damage[3,4,5]. **m)** Protein expression analysis by western blot for genes characteristic for major clusters and with a putative role within the specific biology of identified CLL subtypes. Whole cell protein was isolated from a representative cohort of samples based on availability of patient material (major subtypes / no alterations besides del(13q)); multiple western blots were performed from the lysates to enable validation of RNA expression patterns for basal expression of proteins of interest, along with the corresponding house keeping genes (actin).

Supplementary References

1. Roberts SA, Lawrence MS, Klimczak LJ, et al. An APOBEC cytidine deaminase mutagenesis pattern is widespread in human cancers. *Nat Genet*. 2013;45(9):970-976.
2. Stavrou S, Blouch K, Bass A, Correspondence SRR, Kotla S, Ross SR. Nucleic Acid Recognition Orchestrates the Anti-Viral Response to Retroviruses. *Cell Host Microbe*. 2015;17(4):478-488.
3. Brzostek-Racine S, Gordon C, Van Scoy S, Reich NC. The DNA Damage Response Induces IFN. *J Immunol*. 2011;187(10):5336-45.
4. Kondo T, Kobayashi J, Saitoh T, et al. DNA damage sensor MRE11 recognizes cytosolic double-stranded DNA and induces type I interferon by regulating STING trafficking. *PNAS U S A*. 2013;110(8):2969-2974.
5. Burdette DL, Monroe KM, Sotelo-Troha K, et al. STING is a direct innate immune sensor of cyclic di-GMP. *Nature*. 2011;478(7370):515-518.

Supplementary Figure 4: Overexpression of AID induces genomic instability.

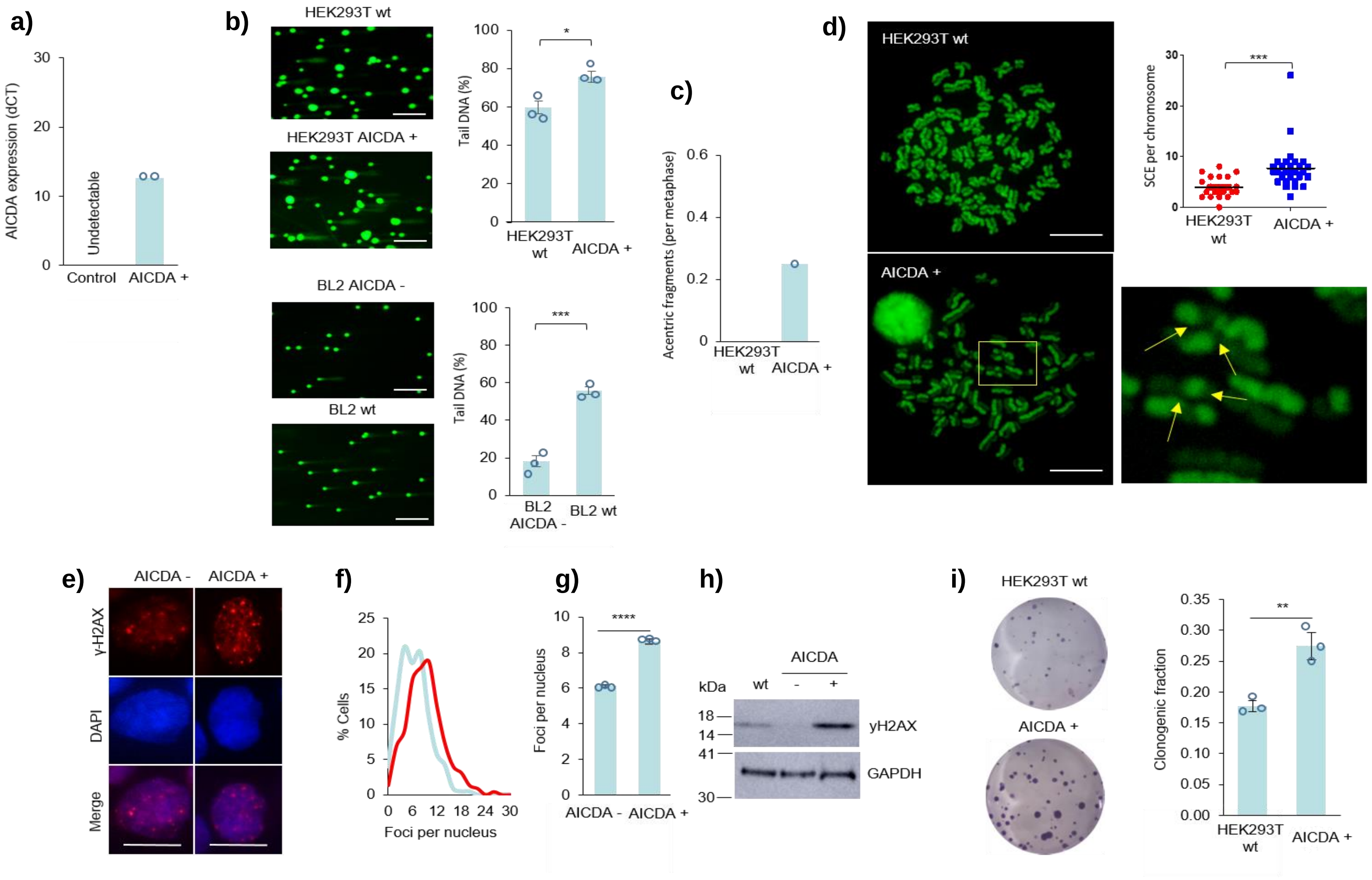

Supplementary Figure 4: Overexpression of AID induces genomic instability.

a) Shown is the qPCR analysis of control vs. *AICDA* overexpressing HEK293T cells after transfection with *AICDA* or empty vector. RNA was isolated using RNAeasy mini kit (Qiagen) and then qPCR reactions were performed using *AICDA* Tagman Hs00757808_m1 probe, accompanied by *ACTB* Hs99999903_m1 and 18S Hs99999901_s1 housekeeping gene probes. **b)** Alkaline single cell electrophoresis (comet) assay for the assessment of steady state single strand DNA breaks in *AICDA*+ vs. HEK293Twt (top panels) or BL2 AID negative mutant (*AICDA*-) vs. BL2wt (bottom panels) cells. Data represent percentage of tail DNA from three independent biological replicates ± s.e.m. *p≤0.02, **p≤0.0005. Type 2, 2-sided Student's t-test. Bars 100 µm. **c)** Average number of acentric chromosome fragments in *AICDA*+ vs. HEK293Twt cells. Chromosome spreads were prepared as described in the materials/methods section and then the proportion of acentrics per metaphase was calculated microscopically using x60 primary magnification. At least five random fields containing at least 20 metaphase spreads were analyzed. **d)** Sister Chromatid Exchange (SCE) assay was performed as indicator of cellular genotoxicity and ongoing mutagenesis in HEK293Twt (top left) vs. *AICDA*+ HEK293T (bottom left) cells. Bars 5 µm. Chromosome spreads were prepared following sequential labelling of sister chromatids with BrdU and staining with Acridine Orange as per Materials and Methods. SCEs (bottom right) per metaphase were quantified microscopically from at least five random fields containing n=23 (HEK293T*wt*) and n=28 (HEK293T*AICDA*) metaphase spreads. Two-tailed Student's t-test was used to calculate the significance of variances between samples (top right). **e-h)** Steady state expression of γ-H2AX protein (a universal marker of DNA damage, including DNA double strand breaks) in *AICDA*- vs. *AICDA*+ BL2 cells. γ-H2AX foci (e) were quantified microscopically in a blind manner and then total distribution of foci per cell (f) or average number of foci per biological replicate (g) were calculated. Results represent total (f) or average (g) three biological replicates ± s.e.m. ****p≤0.00004. Type 2, 2-sided Student's t-test. At least 200 nuclei were analyzed per biological replicate, each containing three technical replicate slides. X60 original magnification, DNA was counterstained with DAPI. Bars 20 µm. (h) Western blot analysis of total γ-H2AX content in wt, *AICDA*- and *AICDA*+ BL2 cells. GAPDH was used as a loading control. Representative analysis of three independent experiments. **i)** Clonogenic survival assays were performed to test the impact of AID on the ability to form a large colony or clone as an *in vitro* assay for reproductive capacity in *AICDA*+ and HEK293Twt cells. 200 cells in logarithmic phase of growth were seeded into 6 well plate and, following 10 day incubation, colonies were stained by crystal violet and analyzed using ImageJ software. Clonogenic fraction was calculated as a ratio between number of colonies and the amount of cells seeded. Data represent average from three independent biological replicates ± s.e.m. **p≤0.007. Type 2, 2-sided Student's t-test.

Supplementary Figure 5: EMT-like gene sets, transcriptional changes in tri(12) CLL, epigenetic modifiers in CLL subtypes.

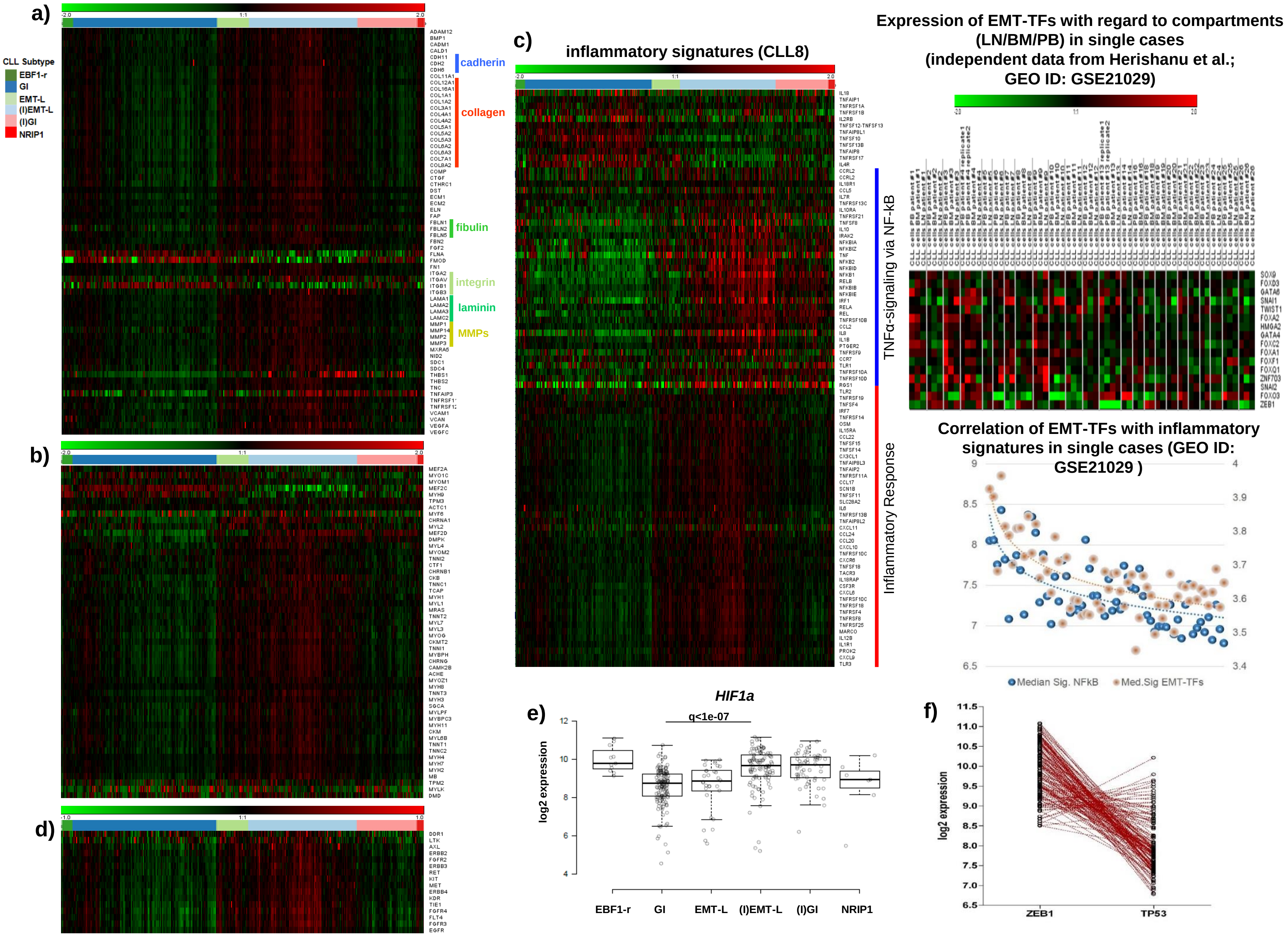

Supplementary Figure 5: EMT-like gene sets, transcriptional changes in tri(12) CLL, epigenetic modifiers in CLL subtypes. Heatmap showing expression profiles of GSEA core enrichment genes. **a)** Genes from the "Epithelial Mesenchymal Transition" signature involved in extracellular matrix remodeling. This process involves extracellular matrix degradation through matrix metalloproteinases (MMPs) and change of the extracellular matrix composition and interaction with collagen, cadherin, fibulin or laminin. CLL subtype color code defined in Supplementary Fig. 5a applies for Supplementary Fig. 5a-d. **b)** Genes associated with contractile actin filament structures and representative for the "Myogenesis" signature (Fig. 2a). Genes contained are for example coding for the myosin light chain family (*MYL*), myosin heavy chain family (*MYH*), troponin complex subunits (*TNN*), creatine kinase (*CK*) and were upregulated in association with other EMT-like features, supporting an increased cell contractility and motility. **c)** Expression of genes representative for "Inflammatory response" and "TNFα-signaling via NF-kB" in CLL8 clusters (left). Expression of EMT-TFs with regard to compartments (LN/BM/PB) in single cases (right top) and correlation of EMT-TFs with inflammatory signatures (right bottom) (independent data set from Herishanu et al.; GEO ID GSE21029). **d)** Expression of receptor tyrosine kinases. **e)** *HIF1α* expression is shown for n=337 biologically independent samples. For the boxplots, centerline, box limits, and whiskers represent the median, 25th, and 75th percentiles and 1.5x interquartile range, respectively. FDR for *HIF1α* from comparison of GI vs. (I)EMT-L is indicated (q<1e-07). **f)** Expression of *ZEB1* and *TP53,* shown for n=233 biologically independent samples (GI and (I)EMT-L cases only), highlights inverse correlation for both genes.

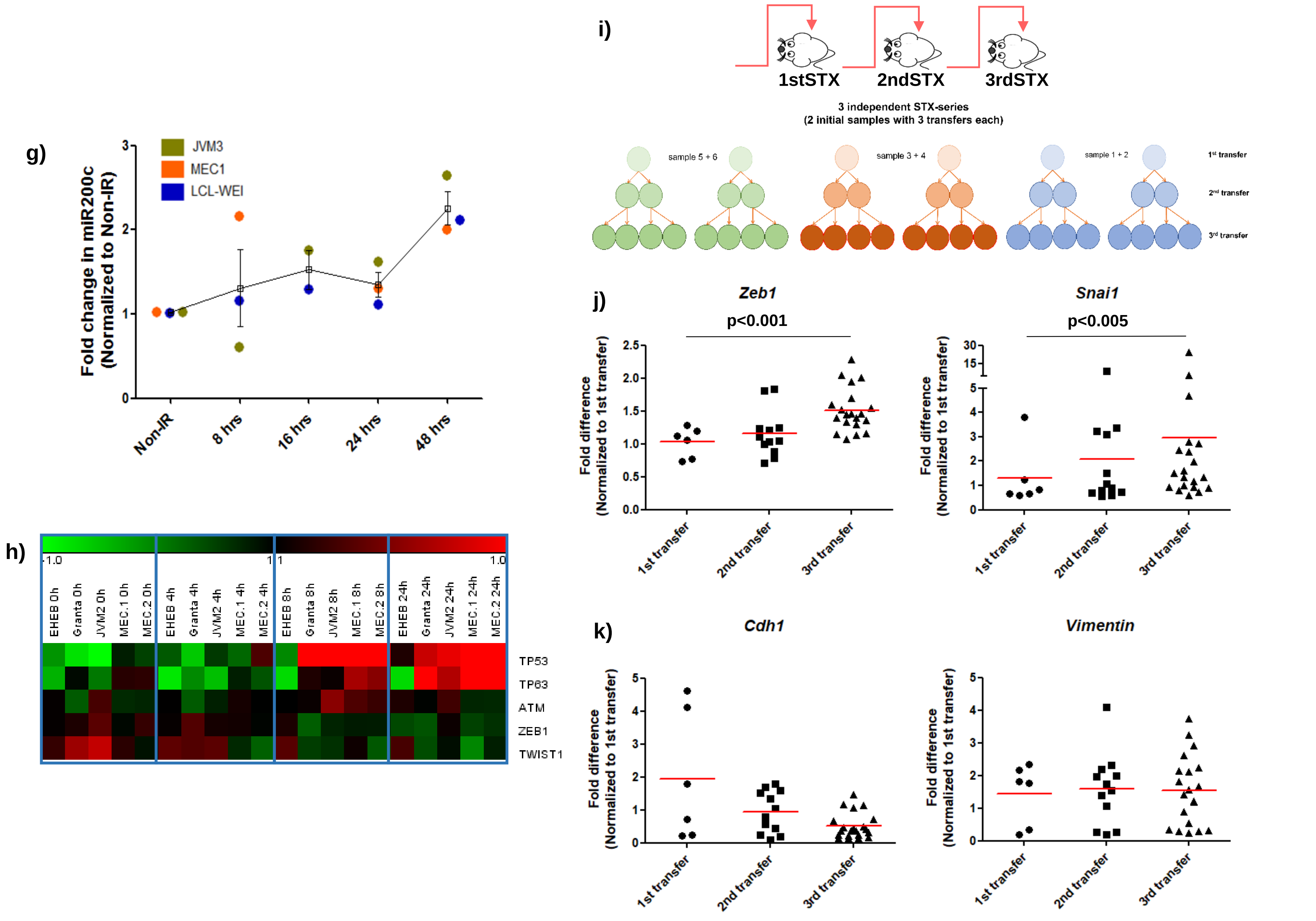

Supplementary Figure 5:

g) Time course of expression (qPCR) for *miR-200c* before irradiation (0h) and at 8h, 24h, 48h after ionizing irradiation with 5Gy shows upregulation in response to DDR/p53 activation. Mean and the standard error of the mean (SEM) are indicated for each time point. Data is shown for n=3 independent cell lines. **h)** Time course of expression (Exon 1.0 Array based assessment, log2 expression scale) shows temporal changes for *TP53*, *TP63*, *ATM*, *ZEB1* and *TWIST1* before (0h) and at 4h, 8h, 24h after ionizing irradiation with 5Gy supporting DNA damage associated downregulation of the *miR-200c* target *ZEB1* and *TWIST1* in different lymphoma cell lines. **i)** Schematic model illustrating serial transplantation of Eμ-TCL1 tumor cells. Samples from 1st transfer were transplanted into 2 recipients each for a total of 3 serial transplantations. In total, three independent series (starting with 2 samples each) were performed. **j)** Figure showing fold difference of expression (qPCR) for *Zeb1* and *Snai1* (from 2nd transfer with n=12 and 3rd transfer with n=20 evaluable measures/samples), mean is indicated by red line. Both *Zeb1* and *Snai1* are significantly upregulated through 2nd and 3rd STX (p<0.001 and <0.005, Friedman rank sum test with Conover p-values (for averaged expression / transfer round in 4 sequential experiments)). **k)** Figure showing fold difference of expression (qPCR) for *Cdh1* and *Vim* (from 2nd transfer with n=12 each and 3rd transfer with n=21 and n=19 evaluable measures/samples), mean is indicated by red line. *Cdh1* is downregulated through 2nd and 3rd STX.

Supplementary Figure 6: Assessment of epigenetic regulation using GEP and DNA methylation analysis.

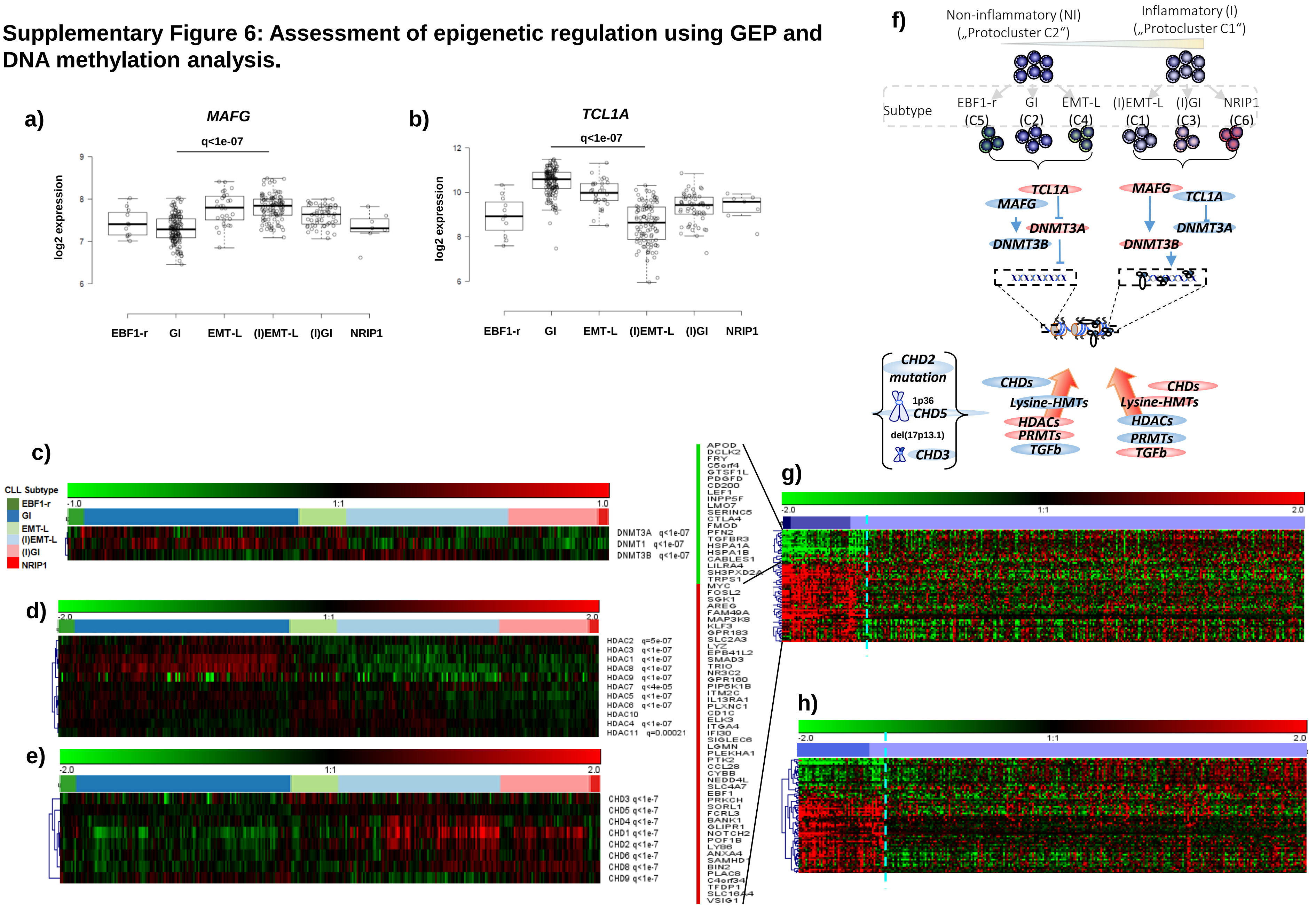

Supplementary Figure 6: Assessment of epigenetic regulation using GEP and DNA methylation analysis.
a) Expression of *MAFG* averaged transcript clusters and **b)** *TCL1A*. For Supplementary Fig. 6a/b, data is shown for n=337 biologically independent samples. For the boxplots, centerline, box limits, and whiskers represent the median, 25th, and 75th percentiles and 1.5x interquartile range, respectively. FDR for *MAFG* and *TCL1A* from comparison of GI vs. (I)EMT-L is indicated (q<1e-07). **c-e)** Heatmaps showing expression of (c) DNA methyltransferases (*DNMT1* functions mainly in maintaining methylation patterns, *DNMT3A* and *DNMT3B* encode de novo DNA methyltransferases), (d) histone deacetylases and (e) chromodomain-helicase-DNA-binding proteins. FDRs of differentially expressed genes for GI vs. (I)EMT-L are indicated on the right (q). CLL subtype color code defined in Supplementary Fig. 6c applies for Supplementary Fig. 6c-e. **f)** Model illustrating CLL subtypes and deregulation/interaction of epigenetic modifiers depicted in Fig. 4d, Supplementary Fig. 3c and Supplementary Fig. 6a-e. Color code indicates mode of regulation (up: red; down: blue) or estimated biological effect (activation: red; inactivation: blue). The transcriptional repressor *MAFG* regulates de novo methylation together with *DNMT3B*. *TCL1A* is a strong inhibitory regulator of the de novo methyltransferase *DNMT3A* in CLL. TGF-β signaling can induce EMT through gene-specific hypermethylation. Expression of arginine and lysine methyltransferases (Fig. 4d, Supplementary Fig. 3c) is associated with the cluster hierarchy and implicates involvement in the regulation of DNA accessibility and epigenetic modulation. Genes coding for chromodomain-helicase-DNA-binding proteins were frequently affected by alterations, including mutations of *CHD2* (Supplementary Fig. 2c), deletion of *CHD3* on chromosome 17p13.1 or specific deregulation of *CHD5* on 1p36 (Supplementary Fig. 3g) and correspondingly altered expression of other *CHDs* (Supplementary Fig. 6e). **g)** Gene set defining tri(12) CLL (as shown in Figure 4l), cases are ordered according to subsets showing 1) healthy B cells (left, dark blue), 2) tri(12) CLL (middle), 3) CLL without tri(12) (light blue). Additionally, all subsets are ordered with decreasing expression levels of *EBF1* from left to right. Cut-off (cyan dotted line) separating CLL without tri(12) but a respective tri(12) profile from all other CLL. **h)** Identical gene set and order for tri(12) and other CLL as used in Supplementary Fig. 6g, shown here for the REACH cohort.

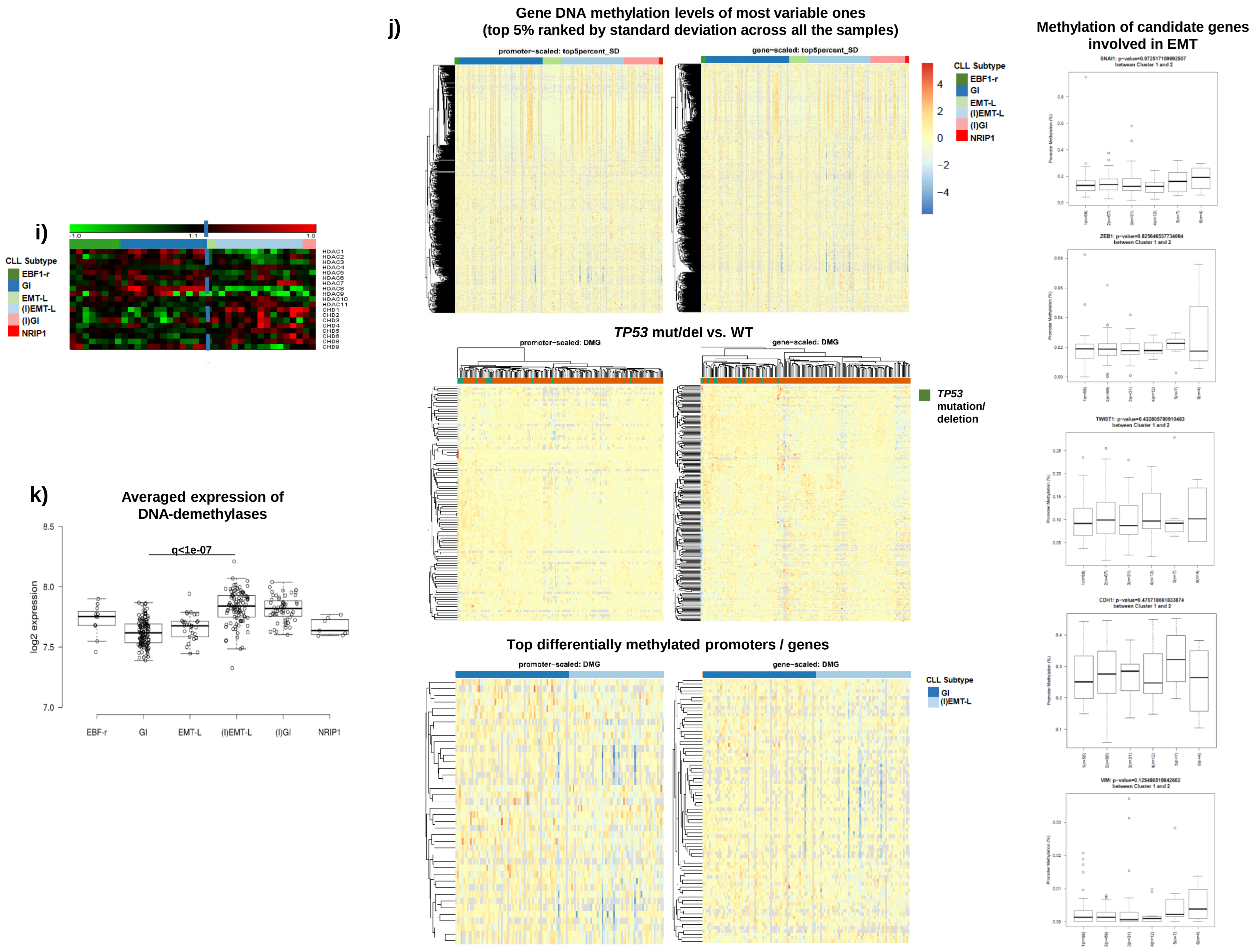

Supplementary Figure 6:

i) Heatmap showing expression of *HDAC* and *CHD* family members in tri(12) cases with regard to respective clusters. **j)** Heatmaps on the top show promoter and gene DNA methylation levels of most variable ones (top 5% ranked by standard deviation across all the samples). Second row heatmaps show promoter and gene DNA methylation levels for cases with *TP53* mutation and/or deletion in comparison to all other cases (*TP53* mutated cases shown in green). Heatmaps on the bottom show specifically assessed methylation differences for promoter and gene DNA methylation levels in (I)EMT-L and GI cases. Only 69 differentially methylated promoters out of 14559 investigated and 130 differentially methylated genes out of 15625 investigated (p<0.05, methylation difference >5%, Mann-Whitney *U*-test (two-sided)) were identified. No promoters or genes were identified as differentially methylated after a Benjamini-Hochberg FDR procedure with BH-FDR <20%. Only promoters or genes with enough coverage (at least 5 covered CpGs for each region) in more than half of patients and non-zero average methylation levels were included into the heatmaps. Data were scaled by row. Box-plots on the right show specific analyses on methylation differences for single genes (*VIM*, *CDH1* and EMT-TFs) involved in EMT-like network regulation. Data is shown for n=182 biologically independent samples, for comparisons of (I)EMT-L (cluster 1) and GI (cluster 2) the t-test (two-sided) was applied. **k)** Averaged expression of DNA-demethylases (e.g. *TET1-3*, other lysine demethylases) in CC subtypes. Data is shown for n=337 biologically independent samples. FDR for averaged demethylase expression from comparison of GI vs. (I)EMT-L is indicated (q<1e-07). For Supplementary Fig. 6j/k the boxplots, centerline, box limits, and whiskers represent the median, 25th, and 75th percentiles and 1.5x interquartile range, respectively.

Supplementary Figure 7: Clinical impact and differential response to treatment in CLL subtypes, CLL8 cohort.

a)

PFS	Pts, N	Events, N	Median months	3-year Survival, %	5-year Survival, %	7-year Survival, %
**(I)EMT-L(C1), GI(C2), (I)GI(C3), EMT-L(C4)**	**319**	**223**				
(I)EMT-L(C1) and FCR *[continuous line]*	48	33	52.0	72.9	42.9	24.3
(I)EMT-L(C1) and FC *[dotted line]*	52	31	36.1	51.8	43.1	31.8
GI(C2) and FCR *[continuous line]*	68	47	42.4	58.6	38.6	27.7
GI(C2) and FC *[dotted line]*	65	53	27.8	34.3	15.0	11.2
(I)GI(C3) and FCR *[continuous line]*	31	18	68.1	71.0	57.6	38.8
(I)GI(C3) and FC *[dotted line]*	25	21	22.8	33.3	17.9	0.0[1]
EMT-L(C4) and FCR *[continuous line]*	15	10	65.2	66.7	53.3	17.5
EMT-L(C4) and FC *[dotted line]*	15	10	45.5	58.7	29.3	29.3[2]

[1] At time point of last observation, month 68.3; [2] month 80.3

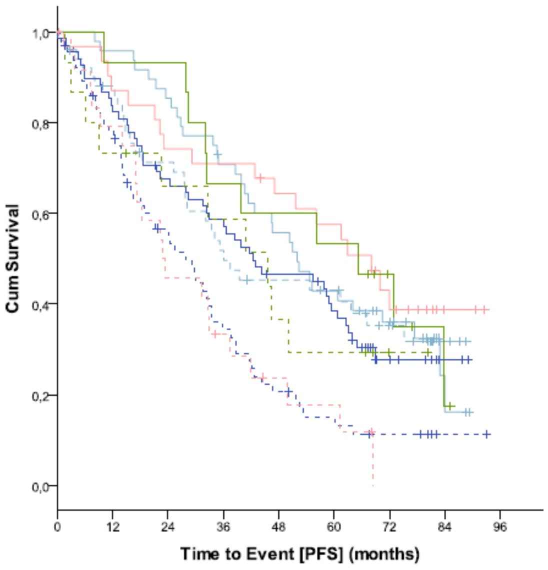

b)

OS	Pts, N	Events, N	Median months	3-year Survival, %	5-year Survival, %	7-year Survival, %
**(I)EMT-L(C1), GI(C2), (I)GI(C3), EMT-L(C4)**	**319**	**117**				
(I)EMT-L(C1) and FCR *[continuous line]*	48	13	NR	95.8	87.1	59.7
(I)EMT-L(C1) and FC *[dotted line]*	52	17	NR	83.8	77.1	56.8
GI(C2) and FCR *[continuous line]*	68	25	NR	82.0	70.6	54.0
GI(C2) and FC *[dotted line]*	65	28	78.9	85.4	68.8	33.8
(I)GI(C3) and FCR *[continuous line]*	31	10	NR	90.3	80.5	58.5
(I)GI(C3) and FC *[dotted line]*	25	13	56.6	66.7	47.9	31.9[1]
EMT-L(C4) and FCR *[continuous line]*	15	5	90.2	100.0	100.0	58.7
EMT-L(C4) and FC *[dotted line]*	15	6	86.3	79.4	65.0	65.0

NR, not reached; [1] At time point of last observation, month 73.8

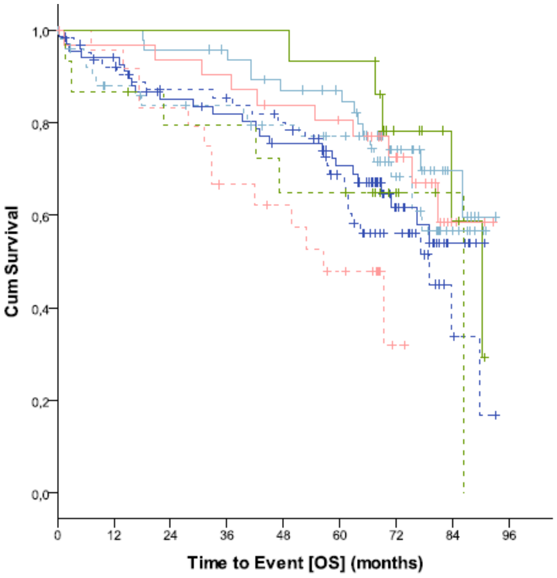

Supplementary Figure 7: Clinical impact and differential response to treatment in CLL subtypes, CLL8 cohort.
a) PFS according to treatment arm and subtype in CLL8 (FC dotted line, FCR continuous line). **b)** OS according to treatment arm and subtype in CLL8 (FC dotted line, FCR continuous line). Groups according to biological subtype are color coded. Median, 3-year, 5-year and 7-year survival times are provided for respective categories in the boxes corresponding to the Kaplan-Meier plots.

Supplementary Figure 8: PFS and OS by IGHV mutation status in CLL subtypes.

a)

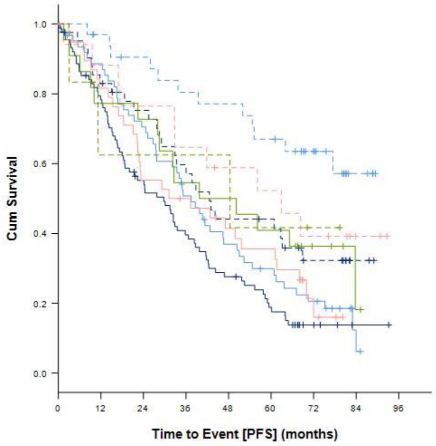

Progression free survival by IgHV *status* and (I)EMT-L(C1), GI(C2), (I)GI(C3), EMT-L(C4)

PFS	Pts, N	Events, N	Median months	3-year Survival, %	5-year Survival, %	7-year Survival, %
***IGHV status* and (I)EMT-L(C1), GI(C2), (I)GI(C3), EMT-L(C4)**	**310**	**218**	**38.8**			
1) (I)EMT-L(C1); *IGHV* unmutated *[continuous line]*	63	50	37.5	50.7	29.9	6.2
2) (I)EMT-L(C1); *IGHV* mutated *[dotted line]*	33	12	NR	80.4	67.0	57.2
3) GI(C2); *IGHV* unmutated *[continuous line]*	88	73	29.8	39.6	18.8	13.8
4) GI(C2); *IGHV* mutated *[dotted line]*	42	26	42.6	59.7	44.1	32.3
5) (I)GI(C3); IGHV unmutated *[continuous line]*	38	29	31.2	50.0	35.6	16.0
6) (I)GI(C3); IGHV mutated *[dotted line]*	18	10	62.9	76.5	52.3	39.2
7) EMT-L(C4) *IGHV* unmutated *[continuous line]*	22	15	39.8	63.6	40.9	18.2
8) EMT-L(C4) *IGHV* mutated *[dotted line]*	6	3	48.4	62.5	41.7	41.7

NR Not reached

b)

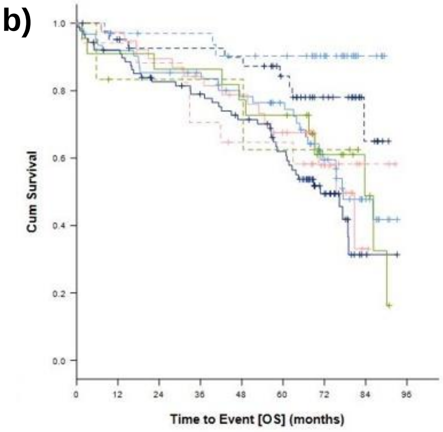

Overall survival by IgHV *status* and (I)EMT-L(C1), GI(C2), (I)GI(C3), EMT-L(C4)

OS	Pts, N	Events, N	Median months	3-year Survival, %	5-year Survival, %	7-year Survival, %
***IGHV status* and (I)EMT-L(C1), GI(C2), (I)GI(C3), EMT-L(C4)**	**310**	**118**	**86.1**			
1) (I)EMT-L(C1); *IGHV* unmutated *[continuous line]*	63	27	77.4	85.3	76.4	47.8
2) (I)EMT-L(C1); *IGHV* mutated *[dotted line]*	33	3	NR	100.0	90.3	90.3
3) GI(C2); *IGHV* unmutated *[continuous line]*	88	43	70.8	79.0	62.0	31.4
4) GI(C2); *IGHV* mutated *[dotted line]*	42	9	NR	92.6	84.3	65.0
5) (I)GI(C3); IGHV unmutated *[continuous line]*	38	16	75.5	84.2	67.5	33.1
6) (I)GI(C3); IGHV mutated *[dotted line]*	18	7	NR	82.4	64.7	58.2
7) EMT-L(C4) *IGHV* unmutated *[continuous line]*	22	11	83.8	86.4	72.7	48.8
8) EMT-L(C4) *IGHV* mutated *[dotted line]*	6	2	NR	83.3	62.5	62.5

NR Not reached

c)

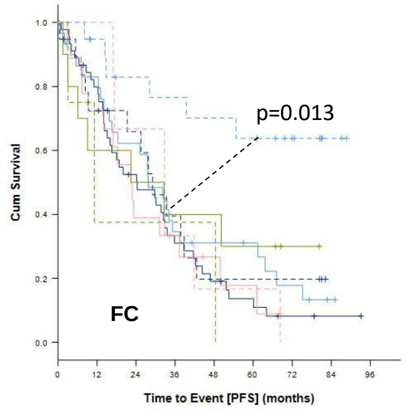

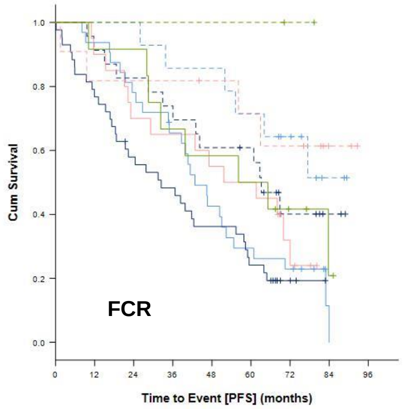

Progression free survival by IgHV status, (I)EMT-L(C1), GI(C2), (I)GI(C3), EM L(C4) and study treatment

Study treatment = FC

PFS	Pts, N	Events, N	Median months	3-year Survival, %	5-year Survival, %	7-year Survival, %
***IGHV status* and (I)EMT-L(C1), GI(C2), (I)GI(C3), EMT-L(C4)**	**153**	**113**	**39.8**			
9) (I)EMT-L(C1); *IGHV* unmutated *[continuous line]*	31	24	27.8	34.6	31.1	13.3
10) (I)EMT-L(C1); *IGHV* mutated *[dotted line]*	19	6	NR	76.5	63.8	63.8
11) GI(C2); *IGHV* unmutated *[continuous line]*	45	39	24.4	31.0	13.6	8.2
12) GI(C2); *IGHV* mutated *[dotted line]*	19	13	29.1	39.5	19.8	19.8
13) (I)GI(C3); IGHV unmutated *[continuous line]*	18	15	22.4	33.3	17.8	8.9
14) (I)GI(C3); IGHV mutated *[dotted line]*	7	6	32.8	66.7	16.7	0.0[1]
15) EMT-L(C4) *IGHV* unmutated *[continuous line]*	10	7	22.5	50.0	30.0	30.0
16) EMT-L(C4) *IGHV* mutated *[dotted line]*	4	3	11.2	37.5	0.0[2]	

NR Not reached; [1] At time point of last observation, month 68.3; [2] month 48.4

Study treatment = FCR

PFS	Pts, N	Events, N	Median months	3-year Survival, %	5-year Survival, %	7-year Survival, %
***IGHV status* and (I)EMT-L(C1), GI(C2), (I)GI(C3), EMT-L(C4)**	**157**	**105**	**54.7**			
9) (I)EMT-L(C1); *IGHV* unmutated *[continuous line]*	32	26	42.8	65.5	29.5	0.0[1]
10) (I)EMT-L(C1); *IGHV* mutated *[dotted line]*	14	6	NR	85.7	71.4	51.4
11) GI(C2); *IGHV* unmutated *[continuous line]*	43	34	32.4	48.3	24.2	19.3
12) GI(C2); *IGHV* mutated *[dotted line]*	23	13	63.2	73.9	60.9	40.1
13) (I)GI(C3); IGHV unmutated *[continuous line]*	20	14	51.7	65.0	50.0	24.0
14) (I)GI(C3); IGHV mutated *[dotted line]*	11	4	NR	81.8	71.6	61.4
15) EMT-L(C4) *IGHV* unmutated *[continuous line]*	12	8	56.2	75.0	50.0	20.8
16) EMT-L(C4) *IGHV* mutated *[dotted line]*	2	0	NR	100.0	100.0	100.0

[1] At time point of last observation, month 84.0
NR Not reached;

d)

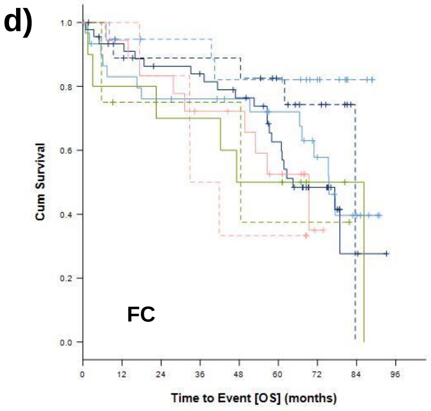

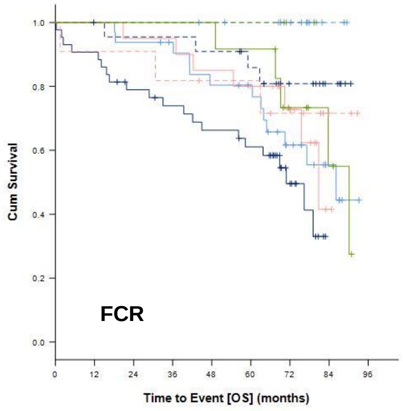

Overall survival by IgHV status, (I)EMT-L(C1), GI(C2), (I)GI(C3), EMT-L(C4) and study treatment

Study treatment = FC

OS	Pts, N	Events, N	Median months	3-year Survival, %	5-year Survival, %	7-year Survival, %
***IGHV status* and (I)EMT-L(C1), GI(C2), (I)GI(C3), EMT-L(C4)**	**153**	**65**	**77.4**			
1) (I)EMT-L(C1); *IGHV* unmutated *[continuous line]*	31	14	75.5	76.0	72.0	39.6
2) (I)EMT-L(C1); *IGHV* mutated *[dotted line]*	19	3	NR	94.7	82.1	82.1
3) GI(C2); *IGHV* unmutated *[continuous line]*	45	22	64.5	83.9	62.6	27.6
4) GI(C2); *IGHV* mutated *[dotted line]*	19	5	83.6	88.9	82.5	0.0[1]
5) (I)GI(C3); IGHV unmutated *[continuous line]*	18	9	69.4	72.2	52.5	35.0
6) (I)GI(C3); IGHV mutated *[dotted line]*	7	4	32.9	83.3	33.3	33.3
7) EMT-L(C4) *IGHV* unmutated *[continuous line]*	10	6	47.2	70.0	50.0	50.0
8) EMT-L(C4) *IGHV* mutated *[dotted line]*	4	2	48.4	75.0	37.5	37.5

NR, not reached; [1] At time point of last observation, month 83.6

Study treatment = FCR

OS	Pts, N	Events, N	Median months	3-year Survival, %	5-year Survival, %	7-year Survival, %
***IGHV status* and (I)EMT-L(C1), GI(C2), (I)GI(C3), EMT-L(C4)**	**157**	**53**	**90.2**			
9) (I)EMT-L(C1); *IGHV* unmutated *[continuous line]*	32	13	86.1	93.8	80.4	55.5
10) (I)EMT-L(C1); *IGHV* mutated *[dotted line]*	14	0	NR	100.0	100.0	100.0
11) GI(C2); *IGHV* unmutated *[continuous line]*	43	21	70.8	73.9	61.1	33.0
12) GI(C2); *IGHV* mutated *[dotted line]*	23	4	NR	95.5	85.9	80.8
13) (I)GI(C3); IGHV unmutated *[continuous line]*	20	7	80.8	95.0	80.0	41.6
14) (I)GI(C3); IGHV mutated *[dotted line]*	11	3	NR	81.8	81.8	71.6
15) EMT-L(C4) *IGHV* unmutated *[continuous line]*	12	5	90.2	100.0	91.7	55.0
16) EMT-L(C4) *IGHV* mutated *[dotted line]*	2	0	NR	100.0	100.0	100.0

NR, not reached;

Supplementary Figure 8: PFS and OS by IGHV mutation status in CLL subtypes, CLL8 cohort.
PFS according to the IGHV mutation status is shown for the 4 major biological subtypes in CLL8 for **a)** all cases / both treatment arms, **c)** the FC and FCR treatment arm separately. OS according to the IGHV mutation status is shown for the 4 major biological subtypes in CLL8 for **b)** all cases / both treatment arms, **d)** the FC and FCR treatment arm separately. Groups according to biological subtype are color coded, dotted lines indicate IGHV mutated and continuous lines IGHV unmutated cases. Median, 3-year, 5-year and 7-year survival times are provided for respective categories in the boxes corresponding to the Kaplan-Meier plots.

Supplementary Figure 9: PFS by (I)EMT-L(C1), GI(C2) and cytogenetics (hierarchical model).

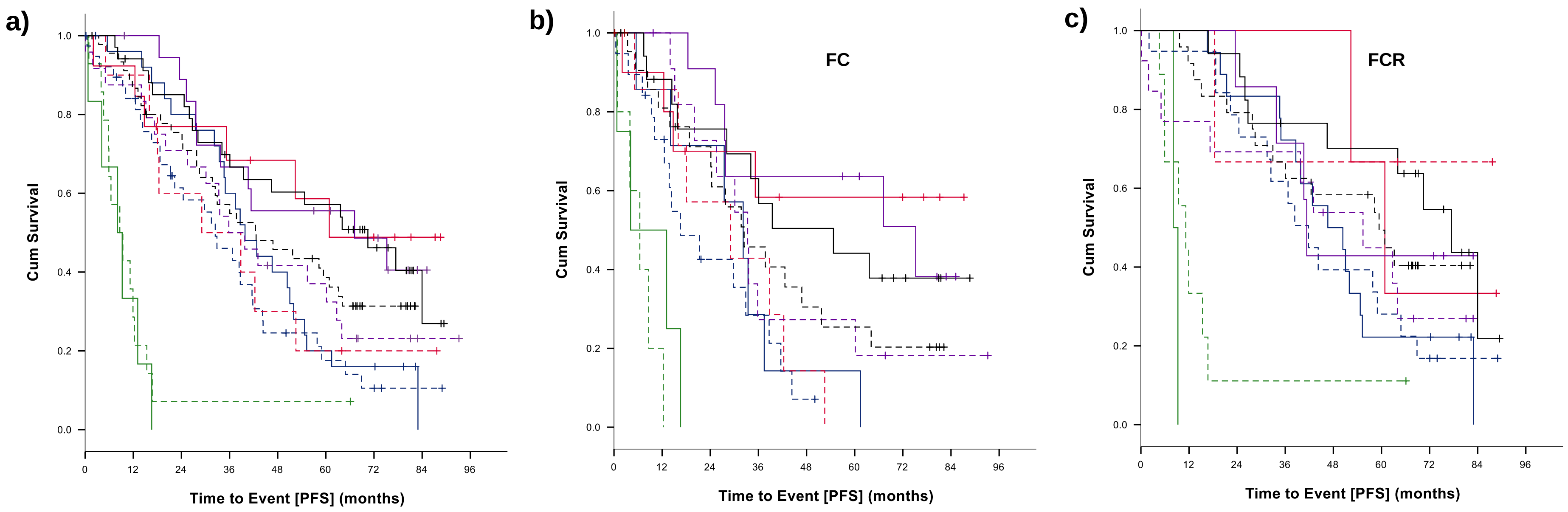

Progression free survival by (I)EMT-L(C1), GI(C2) and cytogenetics

PFS	Pts, N	Events, N	Median months	3-year Survival, %	5-year Survival, %	7-year Survival, %
**(I)EMT-L(C1), GI(C2)**	**231**	**162**				
**(I)EMT-L(C1), del(17p) [continuous line]**	6	6	8.1	0.0[1]	-	-
GI(C2), del(17p) [dotted line]	14	13	8.6	7.1	7.1	7.1[2]
**(I)EMT-L(C1), del(11q) [continuous line]**	27	22	39.9	60.0	20.0	0.0[3]
GI(C2), del(11q) [dotted line]	38	30	32.4	46.0	17.5	10.5
**(I)EMT-L(C1), tri12 [continuous line]**	13	6	60.8	68.40	58.6	48.8
GI(C2), tri12 [dotted line]	10	8	29.1	50.0	20.0	20.0
**(I)EMT-L(C1), normal [continuous line]**	19	10	67.2	66.7	55.6	40.5
GI(C2), normal [dotted line]	24	18	35.8	50.0	37.0	23.1
**(I)EMT-L(C1), del(13q) [continuous line]**	34	19	70.5	69.8	57.1	26.9
GI(C2), del(13q) [dotted line]	46	30	42.6	57.1	38.6	31.4

[1] At time point of last observation, month 16.6; [2] month 66.1; [3] month 83.0

Progression free survival by (I)EMT-L(C1), GI(C2) and cytogenetics, and study treatment

Study treatment = FC

PFS	Pts, N	Events, N	Median months	3-year Survival, %	5-year Survival, %	7-year Survival, %
**(I)EMT-L(C1), GI(C2)**	**116**	**83**				
**(I)EMT-L(C1), del(17p) [continuous line]**	4	4	4.1	0.0[1]	-	-
GI(C2), del(17p) [dotted line]	5	5	6.4	0.0[2]	-	-
**(I)EMT-L(C1), del(11q) [continuous line]**	9	7	32.2	28.6	14.3	0.0[3]
GI(C2), del(11q) [dotted line]	19	15	16.6	28.4	7.1[4]	-
**(I)EMT-L(C1), tri12 [continuous line]**	10	4	NR	58.3	58.3	58.3
GI(C2), tri12 [dotted line]	7	7	29.1	42.9	0.0[5]	-
**(I)EMT-L(C1), normal [continuous line]**	12	6	75.2	63.6	63.6	38.2
GI(C2), normal [dotted line]	11	9	33.3	27.3	27.3	18.2
**(I)EMT-L(C1), del(13q) [continuous line]**	17	10	54.7	63.0	44.1	37.8
GI(C2), del(13q) [dotted line]	22	16	32.4	45.7	25.4	20.3[6]

NR, not reached; [1] At time point of last observation, month 16.6; [2] month 12.3; [3] month 61.4; [4] month 50.1; [5] month 52.5; [6] month 82.2

Study treatment = FCR

PFS	Pts, N	Events, N	Median months	3-year Survival, %	5-year Survival, %	7-year Survival, %
**(I)EMT-L(C1), GI(C2)**	**115**	**79**				
**(I)EMT-L(C1), del(17p) [continuous line]**	2	2	8.1	0.0[1]	-	-
GI(C2), del(17p) [dotted line]	9	8	11.2	11.1	11.1	11.1[2]
**(I)EMT-L(C1), del(11q) [continuous line]**	18	15	46.6	72.2	22.2	0.0[3]
GI(C2), del(11q) [dotted line]	19	15	41.8	61.8	28.1	16.8
**(I)EMT-L(C1), tri12 [continuous line]**	3	2	60.8	100.0	100.0	33.3
GI(C2), tri12 [dotted line]	3	1	NR	66.7	66.7	66.7
**(I)EMT-L(C1), normal [continuous line]**	7	4	41.4	71.4	42.9	42.9[4]
GI(C2), normal [dotted line]	13	9	55.4	69.2	44.9	26.9[5]
**(I)EMT-L(C1), del(13q) [continuous line]**	17	9	77.4	76.5	70.1	21.8
GI(C2), del(13q) [dotted line]	24	14	59.4	66.7	49.4	40.4[6]

NR, not reached; [1] At time point of last observation, month 9.2; [2] month 66.1; [3] month 82.3; [4] month 82.9; [5] month 82.9; [6] month 82.1

Supplementary Figure 9: PFS by (I)EMT-L(C1), GI(C2) and cytogenetics (hierarchical model), CLL8 cohort. PFS according to the hierarchical model for cytogenetic aberrations in the GI and (I)EMT-L subtype in CLL8 for **a)** all cases / both treatment arms, **b)** only the FC treatment arm, **c)** only the FCR treatment arm. Groups according to cytogenetics are color coded, dotted lines indicate association with the GI and continuous lines with the (I)EMT-L subtype. Median, 3-year, 5-year and 7-year survival times are provided for respective categories in the boxes corresponding to the Kaplan-Meier Plots.

Supplementary Figure 10: PFS for GI and (I)EMT-L (*TP53* wild-type) and all cases with *TP53* mutation/deletion.

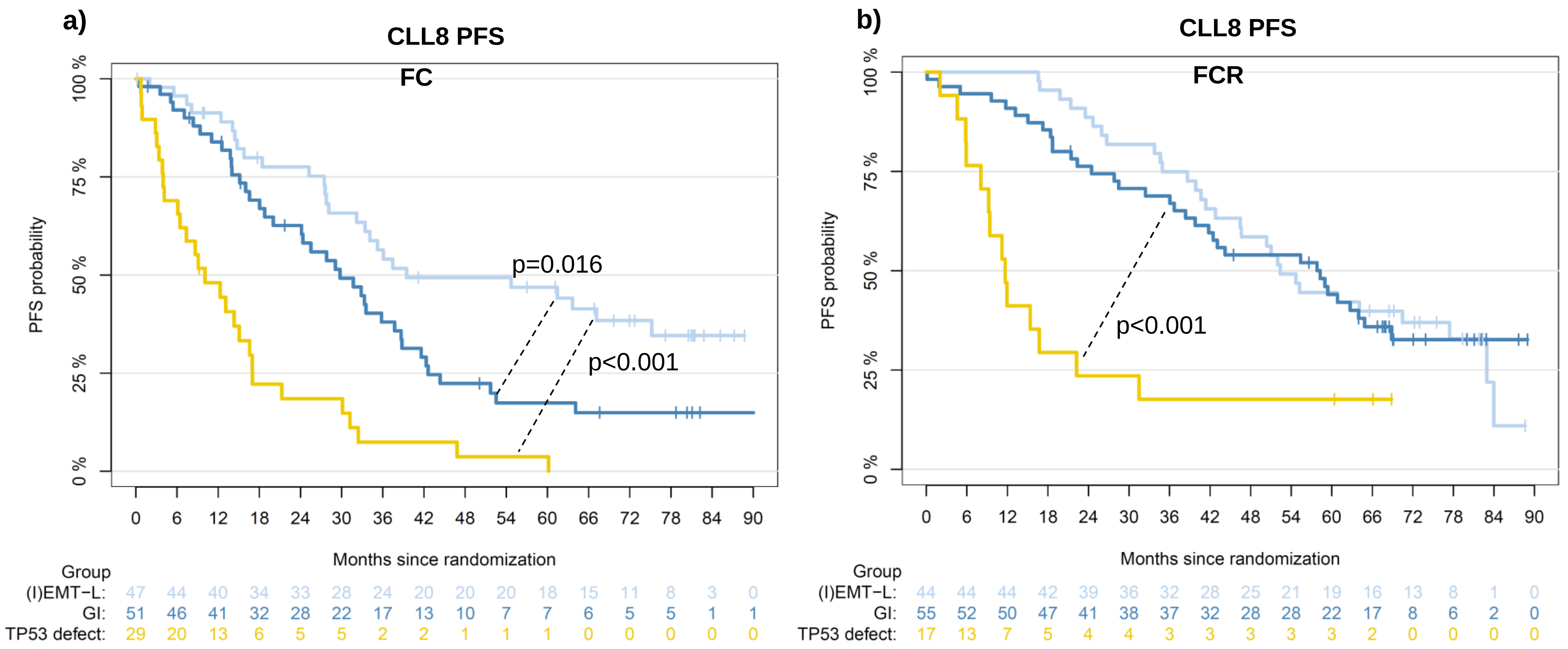

Supplementary Figure 10: PFS for GI and (I)EMT-L (*TP53* wild-type) and all cases with *TP53* mutation/deletion, CLL8 cohort. PFS according to GI subtype with *TP53* wild-type (dark blue), (I)EMT-L subtype with *TP53* wild-type (light blue) and all cases with *TP53* mutation and/or deletion (yellow) in CLL8 for **a)** only the FC treatment arm, **b)** only the FCR treatment arm. Cases without *TP53* defect (irrespective of additional alterations) showed PFS rates at 5 years of 17% in GI vs. 47% in (I)EMT-L (GI: median PFS 29.8 vs. (I)EMT-L: 39.5 months, HR:1.83 (95%CI 1.12-3.0), p=0.016) when treated with FC. The addition of rituximab improved outcome in GI with PFS rates at 5 years of 44%, which were in contrast to 45% at 5 years in (I)EMT-L (GI: median PFS 58.3 months vs. (I)EMT-L: 52.4 months, HR:1.07 (95%CI 0.65-1.74), p=0.79). (I)EMT-L cases therefore lack a similar increase of efficacy for the addition of rituximab when compared to GI.

Supplementary Figure 11: PFS for GI and (I)EMT-L (*TP53/ATM* wild-type), *TP53* mutation and/or deletion and *ATM* mutation and/or deletion.

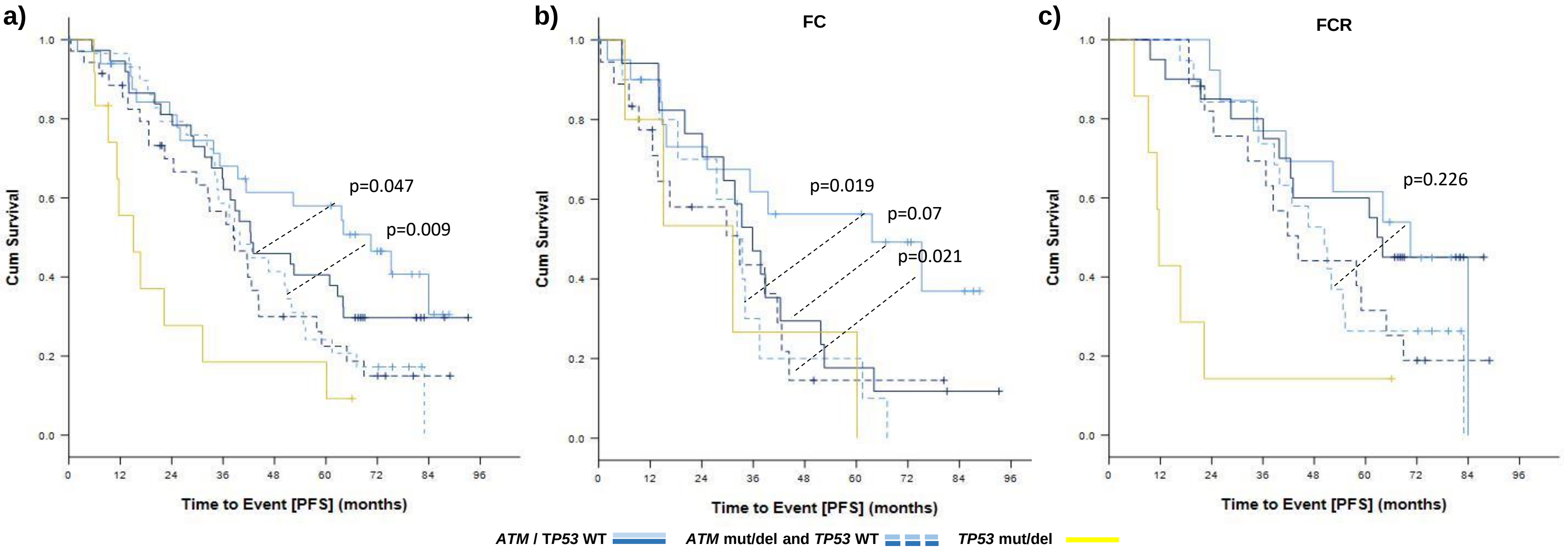

Progression free survival by *ATM mutation/11q Deletion (without TP53 mutation and/or deletion)* and (I)EMT-L(C1), GI(C2)

PFS	Pts, N	Events, N	Median months	3-year Survival, %	5-year Survival, %	7-year Survival, %
***ATM status, TP53 status* and (I)EMT-L(C1), GI(C2)**	**147**	**105**	**41.4**			
1) (I)EMT-L(C1); *ATM and TP53 wild-type [continuous line]*	33	18	70.5	68.0	58.0	30.5
2) (I)EMT-L(C1); *ATM mutation and/or deletion and TP53 wild-type [dotted line]*	30	25	39.9	58.6	24.1	0.0[1]
3) GI(C2); *ATM and TP53 wild-type [continuous line]*	37	26	42.5	64.9	40.5	29.7
4) GI(C2); *ATM mutation and/or deletion and TP53 wild-type [dotted line]*	35	26	38.4	56.6	22.5	15.0
5) All cases with TP53 mutation and/or deletion *[continuous line]*	12	10	15.1	18.5	18.5	9.3

[1] At time point of last observation, month 83.0."Wild-type" specifies that no deletion and or mutation is present.

Progression free survival by *ATM status (without TP53 mutation and/or deletion)*, (I)EMT-L(C1), GI(C2) and study treatment

Study treatment = FC

PFS	Pts, N	Events, N	Median months	3-year Survival, %	5-year Survival, %	7-year Survival, %
***ATM status, TP53 status* and (I)EMT-L(C1), GI(C2)**	**71**	**52**	**34.1**			
6) (I)EMT-L(C1); *ATM and TP53 wild-type [continuous line]*	20	10	63.6	61.9	56.3	36.9
7) (I)EMT-L(C1); *ATM mutation and/or deletion and TP53 wild-type [dotted line]*	11	10	32.2	30.0	20.0	0.0[1]
8) GI(C2); *ATM and TP53 wild-type [continuous line]*	17	15	35.8	47.1	17.6	11.8
9) GI(C2); *ATM mutation and/or deletion and TP53 wild-type [dotted line]*	18	13	32.9	43.5	14.5	14.5
10) All cases with TP53 mutation and/or deletion *[continuous line]*	5	4	31.2	26.7	26.7	0.0[2]

[1] At time point of last observation, month 67.2; [2] month 60.2; "Wild-type" specifies that no deletion and or mutation is present.

Study treatment = FCR

PFS	Pts, N	Events, N	Median months	3-year Survival, %	5-year Survival, %	7-year Survival, %
***ATM status, TP53 status* and (I)EMT-L(C1), GI(C2)**	**76**	**53**	**51.0**			
10) (I)EMT-L(C1); *ATM and TP53 wild-type [continuous line]*	13	8	70.5	76.9	61.5	0.0[1]
11) (I)EMT-L(C1); *ATM mutation and/or deletion and TP53 wild-type [dotted line]*	19	15	50.4	73.7	26.3	0.0[2]
12) GI(C2); *ATM and TP53 wild-type [continuous line]*	20	11	62.7	80.0	60.0	45.0
13) GI(C2); *ATM mutation and/or deletion and TP53 wild-type [dotted line]*	17	13	44.2	69.3	31.5	18.9
14) All cases with TP53 mutation and/or deletion *[continuous line]*	7	6	11.7	14.3	14.3	14.3

[1] At time point of last observation, month 84.0; [2] month 83.0; "Wild-type" specifies that no deletion and or mutation is present.

Supplementary Figure 11: PFS for GI and (I)EMT-L (*TP53/ATM* wild-type), *TP53* and *ATM* mutation and/or deletion, CLL8 cohort. PFS according to the biological subtype GI (dark blue) or (I)EMT-L (light blue) and *TP53* and *ATM* mutation and/or deletion status. Cases with wild-type for both genes are indicated by blue continuous lines, cases with *ATM* mutation and/or deletion but *TP53* wild-type are indicated by blue dotted lines for both biological subtypes, cases with *TP53* mutation and/or deletion are indicated by yellow line. **a)** all cases / both treatment arms, **b)** the FC and **c)** FCR treatment arm. Median, 3-year, 5-year and 7-year survival times are provided for respective categories in the boxes corresponding to the Kaplan-Meier plots.

Supplementary Figure 12: PFS for GI and (I)EMT-L *TP53* wild-type cases by mutation status of *SF3B1*.

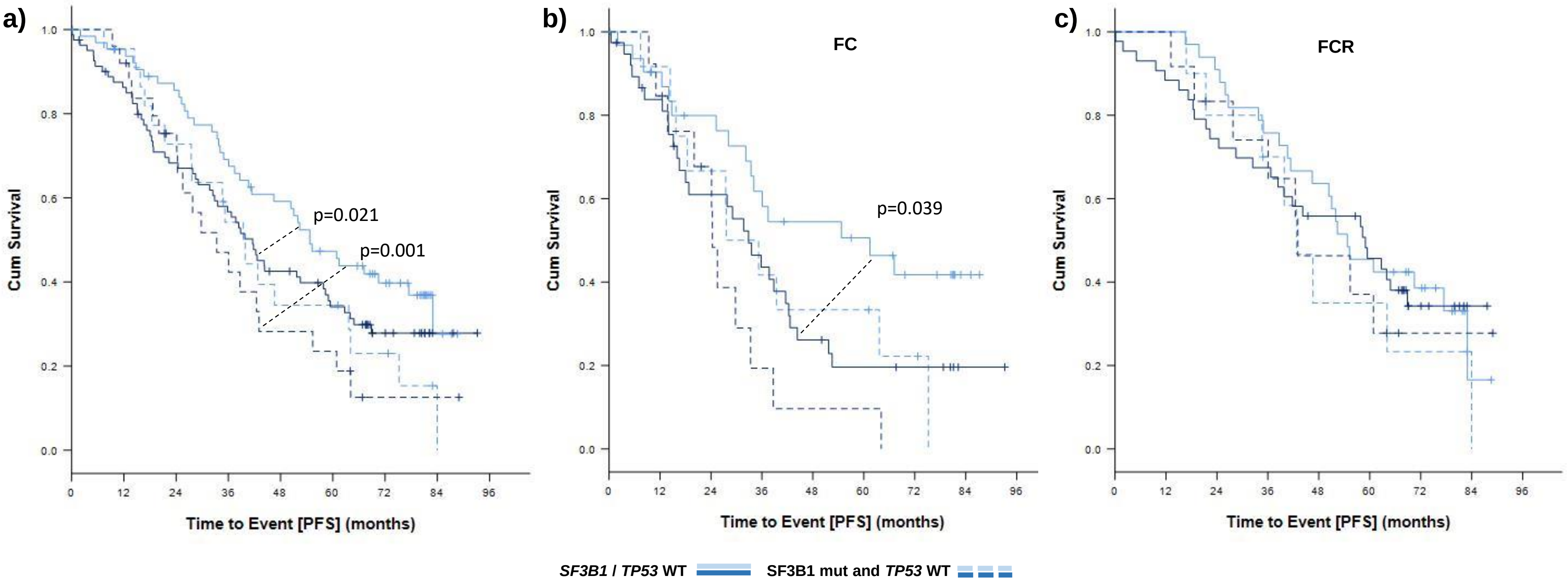

Progression free survival by *SF3B1 status (without TP53 mutation and/(deletion)* and (I)EMT-L(C1), GI(C2)

PFS	Pts, N	Events, N	Median months	3-year Survival, %	5-year Survival, %	7-year Survival, %
***SF3B1* status, *TP53* status and (I)EMT-L(C1), GI(C2)**	**193**	**130**	**42.3**			
1) (I)EMT-L(C1); *SF3B1 and* TP53 wild-type *[continuous line]*	65	38	54.7	69.1	47.3	27.6
2) (I)EMT-L(C1); *SF3B1* mutated and TP53 wild-type *[dotted line]*	22	18	39.5	54.2	34.5	0.0[1]
3) GI(C2); *SF3B1* and TP53 wild-type *[continuous line]*	81	55	41.6	56.7	34.1	27.8
4) GI(C2); *SF3B1* mutated and TP53 wild-type *[dotted line]*	25	19	33.3	47.0	23.5	12.5

[1] At time point of last observation, month 84.0; "Wild-type" specifies that no deletion and/or mutation is present.

Progression free survival by SF3B1 status, TP53 status, (I)EMT-L(C1), GI(C2) and study treatment

Study treatment = FC

PFS	Pts, N	Events, N	Median months	3-year Survival, %	5-year Survival, %	7-year Survival, %
***SF3B1* status, *TP53* status and (I)EMT-L(C1), GI(C2)**	**95**	**65**	**33.5**			
5) (I)EMT-L(C1); *SF3B1 and* TP53 wild-type *[continuous line]*	32	16	61.4	65.4	50.6	41.7
6) (I)EMT-L(C1); *SF3B1* mutated and TP53 wild-type *[dotted line]*	12	10	27.6	41.7	33.3	0.0[1]
7) GI(C2); *SF3B1* and TP53 wild-type *[continuous line]*	38	28	32.9	43.6	19.6	19.6
8) GI(C2); *SF3B1* mutated and TP53 wild-type *[dotted line]*	13	11	24.4	19.3	9.7	0.0[2]

[1] At time point of last observation, month 75.2; [2] month 64.1; "Wild-type" specifies that no deletion and/or mutation is present.

Study treatment = FCR

PFS	Pts, N	Events, N	Median months	3-year Survival, %	5-year Survival, %	7-year Survival, %
***SF3B1* status, *TP53* status and (I)EMT-L(C1), GI(C2)**	**98**	**65**	**55.2**			
1) (I)EMT-L(C1); *SF3B1 and* TP53 wild-type *[continuous line]*	33	22	54.7	75.8	45.5	16.5
2) (I)EMT-L(C1); *SF3B1* mutated and TP53 wild-type *[dotted line]*	10	8	42.8	70.0	35.0	0.0[1]
3) GI(C2); *SF3B1* and TP53 wild-type *[continuous line]*	43	27	59.0	67.4	45.7	34.2
4) GI(C2); *SF3B1* mutated and TP53 wild-type *[dotted line]*	12	8	43.1	74.1	37.0	27.8

[1] At time point of last observation, month 84.0; "Wild-type" specifies that no deletion and/or mutation is present.

Supplementary Figure 12: PFS for GI and (I)EMT-L *TP53* wild-type cases by mutation status of *SF3B1,* CLL8 cohort.
PFS in *TP53* wild-type cases according to the biological subtype GI (dark blue) or (I)EMT-L (light blue) and *SF3B1* mutation status. Cases with wild-type for both genes are indicated by continuous line, cases with *SF3B1* mutation are indicated by dotted line for both biological subtypes.
a) all cases / both treatment arms, **b)** the FC and **c)** FCR treatment arm. Median, 3-year, 5-year and 7-year survival times are provided for respective categories in the boxes corresponding to the Kaplan-Meier plots.

Supplementary Figure 13: Characteristic GEP of CLL subtypes are validated in the REACH cohort.

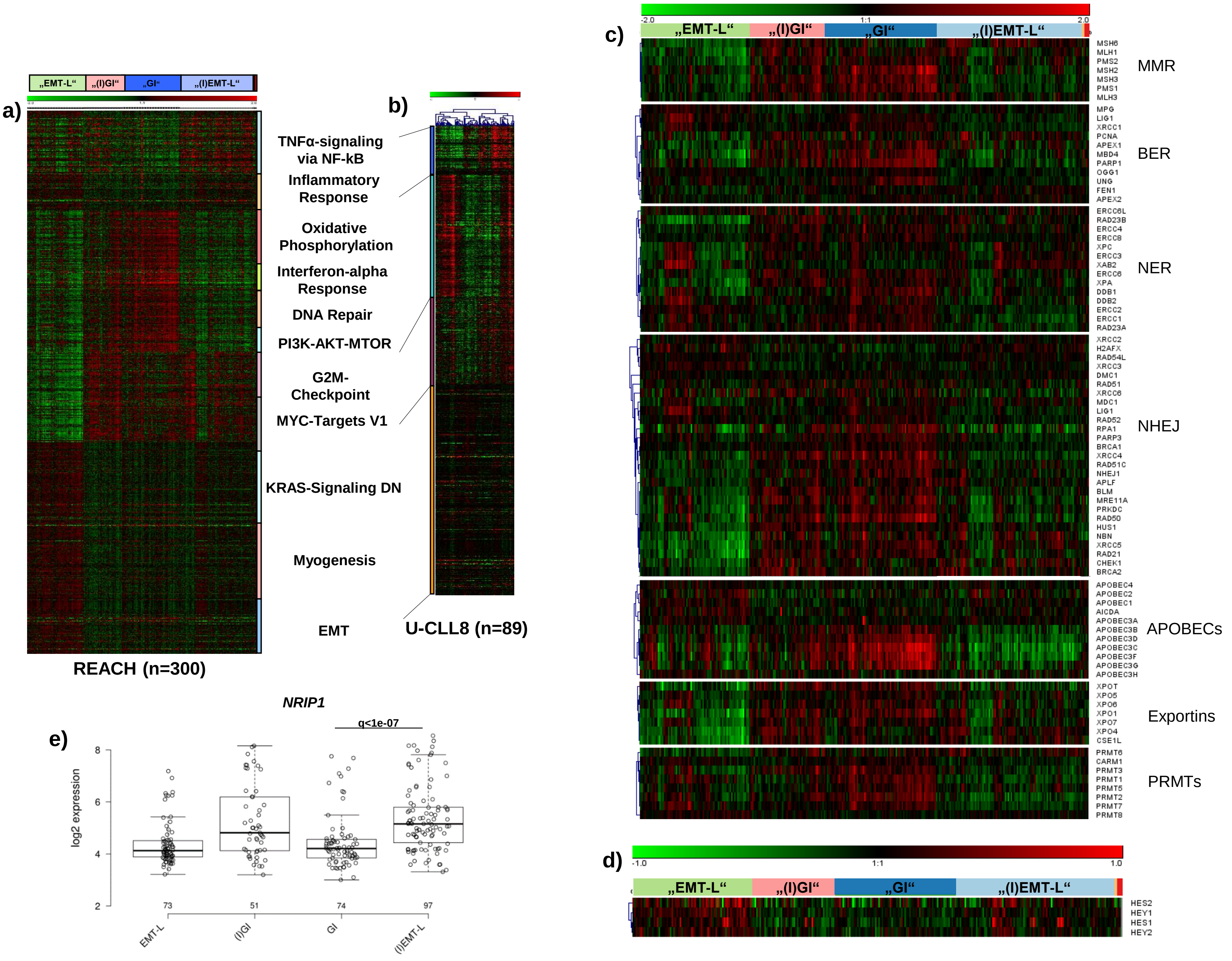

Supplementary Figure 13: Characteristic GEP of CLL subtypes are validated in the REACH cohort.
a) Heatmap showing expression of core enrichment gene sets (as used in Fig. 6b) for the REACH data set. Expression patterns reliably identify identical biologic categories as found in CLL8. Clusters are labeled complementary to CLL8 (indicated by quotation marks). Cases are ordered according to the sequence based on consensus clustering of the REACH data from Fig. 6a. **b)** Internal validation with an independent approach for core gene sets and order (681 genes from the initial signature after repeated filtering for non-variable genes) on unsorted CLL by using hierarchical clustering (Pearson average). **c)** Selected genes from the REACH data set involved in DNA-damage response or resistance (such as NER, BER, *APOBEC* gene family members, etc.) showing distinct overexpression in “GI” and “(I)GI”. Notably, increased expression for these genes in relapsed (REACH) compared to the treatment naive (CLL8) (I)GI cases supports selective pressure on these processes (order of cases is shown as found with consensus clustering in Fig. 6a). **d)** Downstream targets of the NOTCH-pathway *HES1*, *HES2*, *HEY1*, *HEY2* show a switch from enrichment in “(I)EMT-L” to “EMT-L” (order as found with consensus clustering (Fig. 6a)). **e)** *NRIP1* expression with relation to major clusters in the REACH cohort. *NRIP1* overexpression remains strongly associated with inflammatory characteristics which define the (I)GI / (I)EMT-L cluster and shows identical patterns as found in CLL8. Data is shown for n=295 biologically independent samples. For the boxplots, centerline, box limits, and whiskers represent the median, 25th, and 75th percentiles and 1.5x interquartile range, respectively. FDR for *NRIP1* expression from comparison of GI vs. (I)EMT-L is indicated (q<1e-07).

Supplementary Figure 14: Clinical impact of CLL subtypes in relapsed cases, REACH cohort.

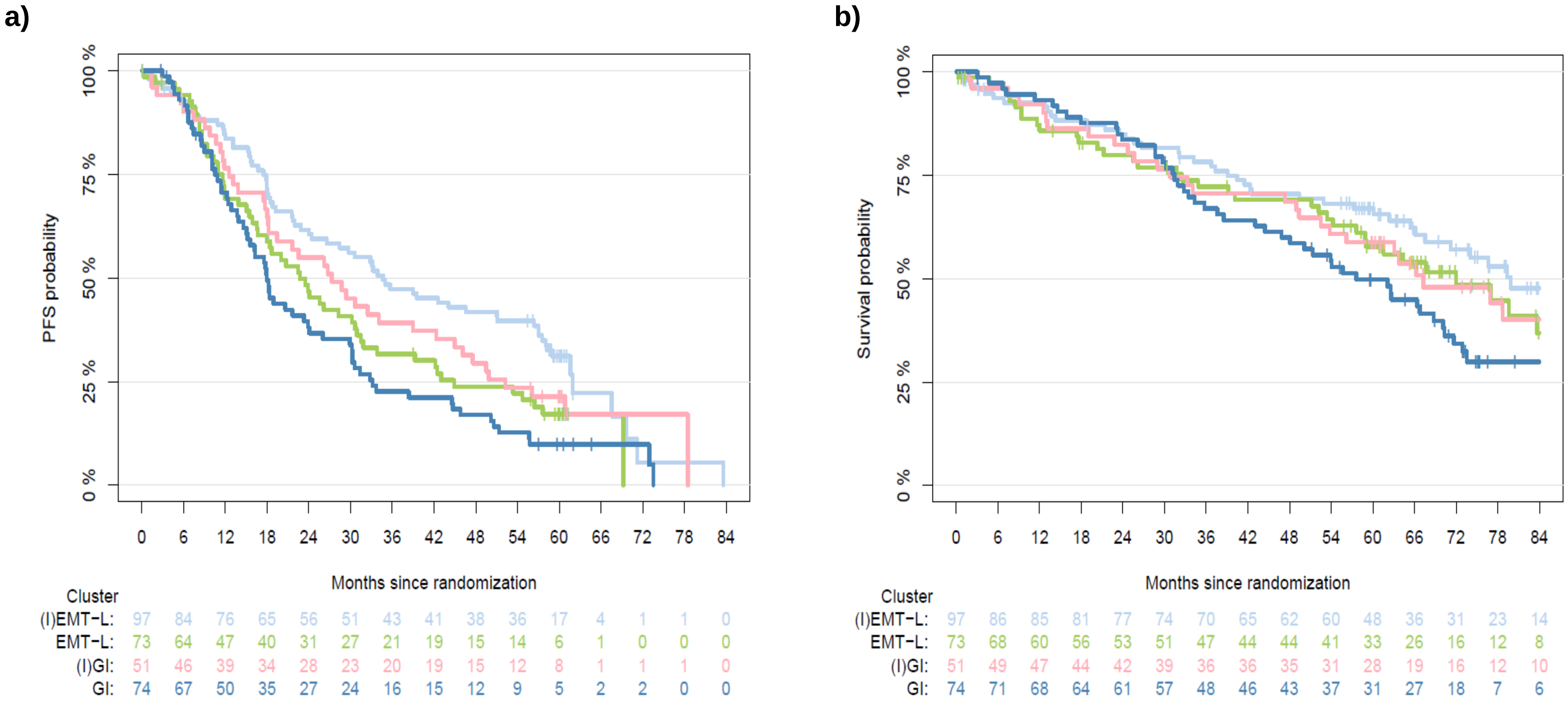

Supplementary Figure 14: Clinical impact of CLL subtypes in relapsed cases, REACH cohort.
a) PFS according to major subtypes in REACH. **b)** OS according to major subtypes in REACH.

Supplementary Tables

Supplementary Table 1)

Patient characteristics for the full CLL8 trial cohort and GEP target analysis population of n=337 CD19 sorted CLL

Baseline characteristics	CD19+ sorted GEP cohort FC treatment	CD19+ sorted GEP cohort FCR treatment	Total CD19+ sorted GEP cohort	CD19+ sorted samples for GEP not available	Total trial cohort
**All patients (ITT), N**	**169**	**168**	**337**	**480**	**817**
**Age at study entry (years)**	**169**	**168**	**337**	**480**	**817**
Median (range)	62 (36-81)	60 (35-77)	61 (35-81)	61 (30-80)	61 (30-81)
**Age group (years), N (%)**	**169**	**168**	**337**	**480**	**817**
≤ 60	77 (45.6)	85 (50.6)	162 (48.1)	236 (49.2)	398 (48.7)
> 60 & ≤ 65	47 (27.8)	45 (26.8)	92 (27.3)	130 (27.1)	222 (27.2)
> 65 & ≤ 70	31 (18.3)	28 (16.7)	59 (17.5)	79 (16.5)	138 (16.9)
> 70	14 (8.3)	10 (6.0)	24 (7.1)	35 (7.3)	59 (7.2)
**Gender, N (%)**	**169**	**168**	**337**	**480**	**817**
Female	41 (24.3)	40 (23.8)	81 (24.0)	129 (26.9)	210 (25.7)
Male	128 (75.7)	128 (76.2)	256 (76.0)	351 (73.1)	607 (74.3)
**Binet stage, N (%)**	**169**	**168**	**337**	**477**	**814**
A	10 (5.9)	11 (6.5)	21 (6.2)	19 (4.0)	40 (4.9)
B	107 (63.3)	99 (58.9)	206 (61.1)	316 (66.2)	522 (64.1)
C	52 (30.8)	58 (34.5)	110 (32.6)	142 (29.8)	252 (31.0)
**ECOG performance status, N (%)**	**159**	**163**	**322**	**463**	**785**
Median (range)	1 (0-1)	0 (0-2)	0 (0-2)	0 (0-2)	0 (0-2)
**Total CIRS score, N (%)**	**169**	**168**	**337**	**479**	**816**
Median (range)	2 (0-7)	2 (0-7)	2 (0-7)	1 (0-8)	1 (0-8)

**Deletion in 17p, N (%)**	**168**	**167**	**335**	**286**	**621**
No	153 (91.1)	154 (92.2)	307 (91.6)	263 (92.0)	570 (91.8)
Yes	15 (8.9)	13 (7.8)	28 (8.4)	23 (8.0)	51 (8.2)
**Deletion in 11q, N (%)**	**168**	**167**	**335**	**286**	**621**
No	125 (74.4)	114 (68.3)	239 (71.3)	229 (80.1)	468 (75.4)
Yes	43 (25.6)	53 (31.7)	96 (28.7)	57 (19.9)	153 (24.6)
**Trisomy 12, N (%)**	**168**	**167**	**335**	**283**	**618**
No	142 (84.5)	155 (92.8)	297 (88.7)	247 (87.3)	544 (88.0)
Yes	26 (15.5)	12 (7.2)	38 (11.3)	36 (12.7)	74 (12.0)
**Deletion in 13q, N (%)**	**168**	**167**	**335**	**282**	**617**
No	65 (38.7)	63 (37.7)	128 (38.2)	139 (49.3)	267 (43.3)
Yes	103 (61.3)	104 (62.3)	207 (61.8)	143 (50.7)	350 (56.7)
**Type according to hier-archical model, N(%)**	**168**	**167**	**335**	**281**	**616**
17p deletion	15 (8.9)	13 (7.8)	28 (8.4)	23 (8.2)	51 (8.3)
11q deletion	39 (23.2)	51 (30.5)	90 (26.9)	52 (18.5)	142 (23.1)
Trisomy 12	21 (12.5)	9 (5.4)	30 (9.0)	31 (11.0)	61 (9.9)
No abnormalities	30 (17.9)	31 (18.6)	61 (18.2)	77 (27.4)	138 (22.4)
13q deletion (single)	63 (37.5)	63 (37.7)	126 (37.6)	98 (34.9)	224 (36.4)
**IGHV mutational status, N (%)**	**163**	**164**	**327**	**295**	**622**
Unmutated	106 (65.0)	109 (66.5)	215 (65.7)	177 (60.0)	392 (63.0)
Mutated	57 (35.0)	55 (33.5)	112 (34.3)	118 (40.0)	230 (37.0)
***TP53* mutational status, N (%)**	**167**	**164**	**331**	**297**	**628**
Unmutated	140 (83.8)	148 (90.2)	288 (87.0)	268 (90.2)	556 (88.5)
Mutated	27 (16.2)	16 (9.8)	43 (13.0)	29 (9.8)	72 (11.5)
***TP53* mutation and/or deletion, N (%)**	**167**	**164**	**331**	**274**	**605**
No	138 (82.6)	147 (89.6)	285 (86.1)	240 (87.6)	525 (86.8)
Yes	29 (17.4)	17 (10.4)	46 (13.9)	34 (12.4)	80 (13.2)

***NOTCH1* mutational status, N (%)**	**163**	**166**	**329**	**293**	**622**
Unmutated	152 (93.3)	149 (89.8)	301 (91.5)	259 (88.4)	560 (90.0)
Mutated	11 (6.7)	17 (10.2)	28 (8.5)	34 (11.6)	62 (10.0)
***SF3B1* mutational status, N (%)**	**163**	**165**	**328**	**293**	**621**
Unmutated	126 (77.3)	130 (78.8)	256 (78.0)	251 (85.7)	507 (81.6)
Mutated	37 (22.7)	35 (21.2)	72 (22.0)	42 (14.3)	114 (18.4)
***ATM* mutational status, N (%)**	**87**	**87**	**174**	**104**	**278**
Unmutated	68 (78.2)	68 (78.2)	136 (78.2)	83 (79.8)	219 (78.8)
Mutated	19 (21.8)	19 (21.8)	38 (21.8)	21 (20.2)	59 (21.2)
***ATM* mutation and/or 11q deletion, N (%)**	**106**	**113**	**219**	**128**	**347**
No	55 (51.9)	57 (50.4)	112 (51.1)	61 (47.7)	173 (49.9)
Yes	51 (48.1)	56 (49.6)	107 (48.9)	67 (52.3)	174 (50.1)
**Telomere length ***	**167**	**166**	**333**	**287**	**620**
Median (range)	4.2 (2.6-11.5)	4.1 (2.6-15.3)	4.2 (2.6-15.3)	5.4 (2.6-28.3)	4.7 (2.6-28.3)
**Telomere length categorical, N (%)**	**167**	**166**	**333**	**287**	**620**
≤ median	114 (68.3)	113 (68.1)	227 (68.2)	83 (28.9)	310 (50.0)
> median	53 (31.7)	53 (31.9)	106 (31.8)	204 (71.1)	310 (50.0)
**Serum thymidine kinase (U/L)**	**148**	**158**	**306**	**285**	**591**
Median (range)	23.4 (3.5-855.0)	17.1 (2.7-970.0)	20.1 (2.7-970.0)	17.4 (2.7-277.0)	18.9 (2.7-970.0)
**Serum thymidine kinase (U/L), N (%)**	**148**	**158**	**306**	**285**	**591**
≤ 10.0	26 (17.6)	38 (24.1)	64 (20.9)	82 (28.8)	146 (24.7)
> 10.0	122 (82.4)	120 (75.9)	242 (79.1)	203 (71.2)	445 (75.3)
**Serum β_2-microglobulin**	**148**	**158**	**306**	**285**	**591**

**(mg/l)**					
Median (range)	2.9 (1.1-9.2)	2.7 (0.9-8.0)	2.8 (0.9-9.2)	2.9 (0.7-10.2)	2.9 (0.7-10.2)

**Serum β_2-microglobulin (mg/l) N (%)**	**148**	**158**	**306**	**285**	**591**
≤ 3.5	99 (66.9)	110 (69.6)	209 (68.3)	188 (66.0)	397 (67.2)
> 3.5	49 (33.1)	48 (30.4)	97 (31.7)	97 (34.0)	194 (32.8)
**Leukocyte count (x 10^9/L)**	**164**	**166**	**330**	**469**	**799**
Median (range)	94.0 (6.7-867.0)	95.6 (12.6-363.0)	94.9 (6.7-867.0)	62.1 (0.2-741.9)	76.8 (0.2-867.0)
**Leukocyte count (x 10^9/L), N (%)**	**164**	**166**	**330**	**469**	**799**
< 50.0	46 (28.0)	37 (22.3)	83 (25.2)	211 (45.0)	294 (36.8)
≥ 50.0	118 (72.0)	129 (77.7)	247 (74.8)	258 (55.0)	505 (63.2)

* median telomere length was defined for full CLL8 trial population

Supplementary Table 2)

Patient characteristics by consensus clustering with k=6 clusters

Baseline characteristics	(I)EMT-L(C1)	GI(C2)	(I)GI(C3)	EMT-L(C4)	EBF1-r(C5)	NRIP1(C6)
**Target analysis population, N**	**100**	**133**	**56**	**30**	**11**	**7**
**Age at study entry (years)**	**100**	**133**	**56**	**30**	**11**	**7**
Median (range)	61 (35-81)	62 (39-77)	58.5 (36-77)	60.5 (40-78)	60 (43-69)	63 (54-74)
**Age group (years), N (%)**	**100**	**133**	**56**	**30**	**11**	**7**
≤ 60	49 (49.0)	58 (43.6)	32 (57.1)	15 (50.0)	6 (54.5)	2 (28.6)
> 60 & ≤ 65	24 (24.0)	38 (28.6)	13 (23.2)	10 (33.3)	3 (27.3)	4 (57.1)
> 65 & ≤ 70	18 (18.0)	28 (21.1)	7 (12.5)	4 (13.3)	2 (18.2)	0 (0.0)
> 70	9 (9.0)	9 (6.8)	4 (7.1)	1 (3.3)	0 (0.0)	1 (14.3)
**Gender, N (%)**	**100**	**133**	**56**	**30**	**11**	**7**
Female	32 (32.0)	27 (20.3)	11 (19.6)	7 (23.3)	3 (27.3)	1 (14.3)
Male	68 (68.0)	106 (79.7)	45 (80.4)	23 (76.7)	8 (72.7)	6 (85.7)
**Binet stage, N (%)**	**100**	**133**	**56**	**30**	**11**	**7**
A	9 (9.0)	8 (6.0)	1 (1.8)	2 (6.7)	1 (9.1)	0 (0.0)
B	59 (59.0)	76 (57.1)	38 (67.9)	21 (70.0=	9 (81.8)	3 (42.9)
C	32 (32.0)	49 (36.8)	17 (30.4)	7 (23.3)	1 (9.1)	4 (57.1)
**ECOG performance status, N (%)**	**92**	**128**	**55**	**30**	**10**	**7**
Median (range)	1 (0-1)	0 (0-2)	0 (0-1)	0 (0-1)	0.5 (0-1)	0 (0-1)
**Total CIRS score, N (%)**	**100**	**133**	**56**	**30**	**11**	**7**
Median (range)	1 (0-6)	2 (0-7)	2 (0-7)	1 (0-5)	2 (0-5)	2 (0-4)
**Deletion in 17p, N (%)**	**99**	**132**	**56**	**30**	**11**	**7**
No	93 (93.9)	118 (89.4)	53 (94.6)	29 (96.7)	7 (63.6)	7 (100.0)
Yes	6 (6.1)	14 (10.6)	3 (5.4)	1 (3.3)	4 (36.4)	0 (0.0)
**Deletion in 11q, N (%)**	**99**	**132**	**56**	**30**	**11**	**7**
No	71 (71.7)	90 (68.2)	40 (71.4)	22 (73.3)	9 (81.8)	7 (100.0)
Yes	28 (28.3)	42 (31.8)	16 (28.6)	8 (26.7)	2 (18.2)	0 (0.0)
**Trisomy 12, N (%)**	**99**	**132**	**56**	**30**	**11**	**7**
No	85 (85.9)	119 (90.2)	54 (96.4)	29 (96.7)	3 (27.3)	7 (100.0)
Yes	14 (14.1)	13 (9.8)	2 (3.6)	1 (3.3)	8 (72.7)	0 (0.0)
**Deletion in 13q, N (%)**	**99**	**132**	**56**	**30**	**11**	**7**
No	43 (43.4)	49 (37.1)	20 (35.7)	7 (23.3)	8 (72.7)	1 (14.3)
Yes	56 (56.6)	83 (62.9)	36 (64.3)	23 (76.7)	3 (27.3)	6 (85.7)
**Type according to hierarchical model, N (%)**	**99**	**132**	**56**	**30**	**11**	**7**
17p deletion	6 (6.1)	14 (10.6)	3 (5.4)	1 (3.3)	4 (36.4)	0 (0.0)
11q deletion	27 (27.3)	38 (28.8)	15 (26.8)	8 (26.7)	2 (18.2)	0 (0.0)
Trisomy 12	13 (13.1)	10 (7.6)	2 (3.6)	0 (0.0)	5 (45.5)	0 (0.0)
No abnormalities	19 (19.2)	24 (18.2)	13 (23.2)	4 (13.3)	0 (0.0)	1 (14.3)
13q deletion (single)	34 (34.3)	46 (34.8)	23 (41.1)	17 (56.7)	0 (0.0)	6 (85.7)
***IGHV* mutational status, N (%)**	**96**	**130**	**56**	**29**	**10**	**6**
Unmutated	63 (65.6)	88 (67.7)	38 (67.9)	22 (75.9)	4 (40.0)	0 (0.0)
Mutated	33 (34.4)	42 (32.3)	18 (32.1)	7 (24.1)	6 (60.0)	6 (100.0)
***TP53* mutational status, N (%)**	**97**	**130**	**56**	**30**	**11**	**7**
Unmutated	92 (94.8)	107 (82.3)	48 (85.7)	28 (93.3)	7 (63.6)	6 (85.7)
Mutated	5 (5.2)	23 (17.7)	8 (14.3)	2 (6.7)	4 (36.4)	1 (14.3)
***TP53* mutation and/or deletion, N (%)**	**97**	**130**	**56**	**30**	**11**	**7**
No	91 (93.8)	106 (81.5)	47 (83.9)	28 (93.3)	7 (63.6)	6 (85.7)
Yes	6 (6.2)	24 (18.5)	9 (16.1)	2 (6.7)	4 (36.4)	1 (14.3)

***NOTCH1* mutational status, N (%)**	**95**	**132**	**56**	**29**	**10**	**7**
Unmutated	90 (94.7)	117 (88.6)	52 (92.9)	25 (86.2)	10 (100.0)	7 (100.0)
Mutated	5 (5.3)	15 (11.4)	4 (7.1)	4 (13.8)	0 (0.0)	0 (0.0)
***SF3B1* mutational status, N (%)**	**95**	**132**	**55**	**29**	**10**	**7**
Unmutated	71 (74.7)	99 (75.0)	42 (76.4)	27 (93.1)	10 (100.0)	7 (100.0)
Mutated	24 (25.3)	33 (25.0)	13 (23.6)	2 (6.9)	0 (0.0)	0 (0.0)
**Telomere length**	**98**	**132**	**56**	**29**	**11**	**7**
Median (range)	4.3 (2.6-10.6)	3.8 (2.8-10.5)	4.1 (2.8-15.3)	4.1 (2.6-9.6)	4.4 (2.9-11.5)	7.4 (4.4-10.8)
**Telomere length categorical, N (%)**	**98**	**132**	**56**	**29**	**11**	**7**
≤ median	58 (59.2)	101 (76.5)	41 (73.2)	20 (69.0)	6 (54.5)	1 (14.3)
> median	40 (40.8)	31 (23.5)	15 (26.8)	9 (31.0)	5 (45.5)	6 (85.7)
**Serum thymidine kinase (U/L)**	**85**	**125**	**49**	**29**	**11**	**7**
Median (range)	17.8 (2.8-855.0)	22.0 (3.2-970.0)	16.9 (3.5-213.0)	23.5 (4.2-199.0)	20.8 (9.5-289.0)	10.9 (2.7-84.6)
**Serum thymidine kinase (U/L), N (%)**	**85**	**125**	**49**	**29**	**11**	**7**
≤ 10.0	22 (25.9)	25 (20.0)	9 (18.4)	5 (17.2)	1 (9.1)	2 (28.6)
> 10.0	63 (74.1)	100 (80.0)	40 (81.6)	24 (82.8)	10 (90.9)	5 (71.4)
**Serum β_2-microglobulin (mg/L), N (%)**	**85**	**125**	**49**	**29**	**11**	**7**
≤ 3.5	65 (76.5)	82 (65.6)	35 (71.4)	16 (55.2)	6 (54.5)	5 (71.4)
> 3.5	20 (23.5)	43 (34.4)	14 (28.6)	13 (44.8)	5 (45.5)	2 (28.6)
**Leukocyte count (x 10^9/L)**	**96**	**132**	**55**	**29**	**11**	**7**
Median (range)	61.1 (8.6-301)	123.5 (17.7-498)	82.2 (12.3-316)	173 (39.3-490.5)	69.80 (14.6-148.3)	103.96 (18.1-228.0)

Supplementary Table 3) ZAP-70 and V-gene usage in k=6 clusters

Baseline characteristics		(I)EMT-L(C1)		GI(C2)		(I)GI(C3)		EMT-L(C4)		EBF1-r(C5)		NRIP1(C6)			
ZAP70 positive	no	34	75.6	57	58.2	19	54.3	7	46.7	7	87.5	4	100.0	128	62.4
	yes	11	24.4	41	41.8	16	45.7	8	53.3	1	12.5	0	0.0	77	37.6
	all	45	100.0	98	100.0	35	100.0	15	100.0	8	100.0	4	100.0	205	100.0
VH gene		4	4.0	3	2.3	0	0.0	1	3.3	1	9.1	1	14.3	10	3.0
	DP-71	1	1.0	0	0.0	0	0.0	0	0.0	0	0.0	0	0.0	1	0.3
	DP58-	0	0.0	1	0.8	0	0.0	0	0.0	0	0.0	0	0.0	1	0.3
	V1-02	3	3.0	6	4.5	3	5.4	1	3.3	0	0.0	0	0.0	13	3.9
	V1-03	2	2.0	5	3.8	0	0.0	1	3.3	0	0.0	0	0.0	8	2.4
	V1-08	1	1.0	1	0.8	0	0.0	0	0.0	0	0.0	0	0.0	2	0.6
	V1-18	0	0.0	0	0.0	1	1.8	0	0.0	0	0.0	0	0.0	1	0.3
	V1-24	0	0.0	1	0.8	0	0.0	1	3.3	1	9.1	0	0.0	3	0.9
	V1-46	2	2.0	1	0.8	1	1.8	0	0.0	0	0.0	0	0.0	4	1.2
	V1-69	21	21.0	31	23.3	12	21.8	6	20.0	1	9.1	0	0.0	71	21.1
	V1-E	0	0.0	1	0.8	0	0.0	0	0.0	0	0.0	0	0.0	1	0.3
	V2-05	1	1.0	1	0.8	0	0.0	0	0.0	1	9.1	0	0.0	3	0.9
	V2-10	1	1.0	0	0.0	0	0.0	0	0.0	0	0.0	0	0.0	1	0.3
	V2-70	0	0.0	1	0.8	0	0.0	0	0.0	0	0.0	1	14.3	2	0.6
	V3-07	1	1.0	3	2.3	1	1.8	1	3.3	0	0.0	0	0.0	6	1.8
	V3-09	2	2.0	2	1.5	3	5.4	2	6.7	0	0.0	0	0.0	9	2.7
	V3-11	9	9.0	4	3.0	3	5.4	0	0.0	1	9.1	0	0.0	17	5.1
	V3-13	0	0.0	0	0.0	1	1.8	0	0.0	0	0.0	1	14.3	2	0.6
	V3-15	0	0.0	4	3.0	0	0.0	1	3.3	0	0.0	0	0.0	5	1.5
	V3-21	1	1.0	4	3.0	7	12.7	1	3.3	0	0.0	0	0.0	13	3.9
	V3-23	8	8.0	5	3.8	3	5.4	1	3.3	0	0.0	0	0.0	17	5.1
	V3-30	6	6.0	7	5.3	2	3.6	1	3.3	0	0.0	0	0.0	16	4.8
	V3-33	6	6.0	6	4.5	3	5.4	2	6.7	0	0.0	0	0.0	17	5.1
	V3-34	0	0.0	1	0.8	0	0.0	0	0.0	0	0.0	0	0.0	1	0.3
	V3-38	0	0.0	0	0.0	1	1.8	0	0.0	0	0.0	0	0.0	1	0.3
	V3-43	2	2.0	1	0.8	0	0.0	1	3.3	0	0.0	0	0.0	4	1.2
	V3-48	4	4.0	6	4.5	3	5.4	0	0.0	0	0.0	0	0.0	13	3.9
	V3-49	0	0.0	1	0.8	0	0.0	0	0.0	1	9.1	0	0.0	2	0.6
	V3-53	0	0.0	2	1.5	0	0.0	0	0.0	1	9.1	0	0.0	3	0.9
	V3-64	1	1.0	0	0.0	1	1.8	1	3.3	0	0.0	0	0.0	3	0.9
	V3-65	1	1.0	0	0.0	0	0.0	0	0.0	0	0.0	0	0.0	1	0.3
	V3-66	1	1.0	1	0.8	1	1.8	1	3.3	0	0.0	0	0.0	4	1.2
	V3-72	0	0.0	1	0.8	1	1.8	1	3.3	0	0.0	0	0.0	3	0.9
	V3-73	0	0.0	0	0.0	1	1.8	0	0.0	1	9.1	0	0.0	2	0.6
	V3-74	3	3.0	5	3.8	0	0.0	0	0.0	0	0.0	2	28.6	10	3.0
	V3-D	1	1.0	1	0.8	0	0.0	0	0.0	0	0.0	0	0.0	2	0.6
	V4-04	0	0.0	2	1.5	1	1.8	1	3.3	0	0.0	0	0.0	4	1.2
	V4-30	0	0.0	2	1.5	0	0.0	1	3.3	1	9.1	0	0.0	4	1.2
	V4-31	1	1.0	0	0.0	0	0.0	0	0.0	0	0.0	1	14.3	2	0.6
	V4-34	4	4.0	11	8.3	2	3.6	2	6.7	0	0.0	0	0.0	19	5.6
	V4-39	5	5.0	2	1.5	0	0.0	0	0.0	0	0.0	0	0.0	7	2.1
	V4-59	1	1.0	1	0.8	2	3.6	1	3.3	0	0.0	0	0.0	5	1.5
	V4-61	1	1.0	1	0.8	1	1.8	1	3.3	0	0.0	0	0.0	4	1.2
	V4-B	1	1.0	2	1.5	0	0.0	0	0.0	0	0.0	0	0.0	3	0.9
	V4B /	0	0.0	1	0.8	0	0.0	0	0.0	0	0.0	0	0.0	1	0.3
	V5-51	2	2.0	2	1.5	0	0.0	1	3.3	1	9.1	1	14.3	7	2.1
	V5-A	1	1.0	0	0.0	1	1.8	0	0.0	0	0.0	0	0.0	2	0.6
	V6-01	1	1.0	1	0.8	0	0.0	0	0.0	1	9.1	0	0.0	3	0.9
	V7-4.	1	1.0	0	0.0	0	0.0	0	0.0	0	0.0	0	0.0	1	0.3
	VH3-8	0	0.0	2	1.5	0	0.0	0	0.0	0	0.0	0	0.0	2	0.6
	all	100	100.0	133	99.9	55	100.0	30	100.0	11	100.0	7	100.0	336	100.0

Supplementary Table 4) Efficacy - Response to treatment for k=6 clusters

	(I)EMT-L(C1) N (%)	GI(C2) N (%)	(I)GI(C3) N (%)	EMT-L(C4) N (%)	EBF1-r(C5) N (%)	NRIP1(C6) N (%)
**All patients**	**100**	**133**	**56**	**30**	**11**	**7**
**Missing response**	8 (8.0)	8 (6.0)	5 (8.9)	1 (3.3)	2 (18.2)	0 (0.0)
**Response**	**92**	**125**	**51**	**29**	**9**	**7**
CR	35 (38.0)	39 (31.2)	20 (39.2)	8 (27.6)	2 (22.2)	2 (28.6)
PR	53 (57.6)	73 (58.4)	26 (51.0)	19 (65.5)	6 (66.7)	4 (57.1)
No response	4 (4.3)	13 (10.4)	5 (9.8)	2 (6.9)	1 (11.1)	1 (14.3)

Supplementary Table 5)

Treatment response according to *TP53* defect, (I)EMT-L(C1) and GI(C2) cluster

**Study treatment FC**	**(I)EMT-L(C1); no *TP53* mutation/ deletion** N (%)	**GI(C2); no *TP53* mutation/ deletion** N (%)	***TP53* mutation/ deletion** N (%)	**p value** (Pearson (two-sided))
**All patients**	**47**	**51**	**29**	
**Missing response**	5 (10.6)	1 (2.0)	7 (24.1)	
**Response**	**42**	**50**	**22**	
CR	11 (26.2)	13 (26.0)	0 (0.0)	< 0.001
PR	29 (60.0)	32 (64.0)	10 (45.5)	
No response	2 (4.8)	5 (10.0)	12 (54.5)	
**Study treatment FCR**	**(I)EMT-L(C1); no *TP53* mutation/deletion** N (%)	**GI(C2); no *TP53* mutation/deletion** N (%)	***TP53* mutation/deletion** N (%)	**p value** (Pearson (two-sided))
**All patients**	**44**	**55**	**17**	
**Missing response**	1 (2.3)	2 (3.6)	2 (11.8)	
**Response**	**43**	**53**	**15**	
CR	22 (51.2)	25 (47.2)	1 (6.7)	< 0.001
PR	21 (48.8)	28 (52.8)	9 (60.0)	
No response	0 (0.0)	0 (0.0)	5 (33.3)	

Supplementary Table 6) Distribution of deletions/mutations of *TP53*, *ATM*, *SF3B1* for GI and (I)EMT-L with regard to IGHV status

	**(I)EMT-L(C1)**		**GI(C2)**		**Total**		**p value** (Pearson (two-sided))
**IGHV status**	**Unmutated**	**Mutated**	**Unmutated**	**Mutated**	**Unmutated**	**Mutated**	
***TP53* mutation and/or 17p deletion, N (%)**	**62**	**32**	**88**	**40**	**150**	**72**	
No	56 (90.3)	32 (100.0)	65 (73.9)	39 (97.5)	121 (80.7)	71 (98.6)	< 0.001
**Yes**	**6 (9.7)**	**0 (0.0)**	**23 (26.1)**	**1 (2.5)**	**29 (19.3)**	**1 (1.4)**	
***ATM mutation and/or 11q deletion*, N (%)**	**45**	**20**	**59**	**29**	**104**	**49**	
No	17 (37.8)	16 (80.0)	25 (42.4)	18 (62.1)	42 (40.4)	34 (69.4)	0.005
**Yes**	**28 (62.2)**	**4 (20.0)**	**34 (57.6)**	**11 (37.9)**	**62 (59.6)**	**15 (30.6)**	
***SF3B1 mutation*, N (%)**	**61**	**31**	**88**	**42**	**149**	**73**	
No	42 (68.9)	26 (83.9)	66 (75.0)	31 (73.8)	108 (72.5)	57 (78.1)	0.482
**Yes**	**19 (31.1)**	**5 (16.1)**	**22 (25.0)**	**11 (26.2)**	**41 (27.5)**	**16 (21.9)**	

Supplementary Table 7)

PFS according to *TP53* defect and cluster assignment to (I)EMT-L(C1) and GI(C2)

Study treatment = FC

PFS	Pts, N	Events, N	Median months	3-year Survival, %	5-year Survival, %	7-year Survival, %
***TP53* and (I)EMT-L(C1), GI(C2)**	**127**	**94**				
1) (I)EMT-L(C1); no *TP53* mutation/ deletion	47	27	39.5	56.4	46.9	34.6
2) GI(C2); no *TP53* mutation/deletion	51	39	29.8	38.0	17.4	14.9
3) *TP53* mutation/deletion	29	28	10.1	7.4	3.7	0.0[1]

[1] At time point of last observation, month 60.2

Study treatment = FCR

PFS	Pts, N	Events, N	Median months	3-year Survival, %	5-year Survival, %	7-year Survival, %
***TP53* and (I)EMT-L(C1), GI(C2)**	**116**	**79**				
4) (I)EMT-L(C1); no *TP53* mutation/ deletion	44	30	52.4	74.9	44.5	11.0
5) GI(C2); no *TP53* mutation/deletion	55	35	58.3	68.8	44.0	32.6
6) *TP53* mutation/deletion	17	14	11.7	17.6	17.6	17.6[1]

[1] At time point of last observation, month 68.8

Supplementary Table 8) Incidence of lethal sepsis in CLL subtypes

	(I)EMT-L (C1)	GI (C2)	(I)GI (C3)	EMT-L (C4)	EBF1-r (C5)	NRIP1 (C6)
Death reason (preferred term), N	4	12	4	2	1	0
BACTERIAL SEPSIS	0	1	0	0	0	0
PULMONARY SEPSIS	1	1	1	0	0	0
SEPSIS	1	8	3	2	1	0
SEPTIC SHOCK	2	2	0	0	0	0

Supplementary Table 9)

Frequency of genetic variables per cluster in the REACH validation cohort

Variable	Levels	„(I)EMT-L“		„EMT-L“		„(I)GI“		„GI“		n_5	$\%_5$	n_6	$\%_6$	n_{all}	$\%_{all}$
TP53 mutation	no	80	92.0	61	91.0	42	89.4	56	81.2	3	100.0	1	100.0	243	88.7
	yes	7	8.1	6	9.0	5	10.6	13	18.8	0	0.0	0	0.0	31	11.3
	all	87	100.0	67	100.0	47	100.0	69	100.0	3	100.0	1	100.0	274	100.0
IGHV unmutated	yes	46	47.9	46	65.7	30	58.8	53	72.6	3	100.0	2	100.0	180	61.0
	no	50	52.1	24	34.3	21	41.2	20	27.4	0	0.0	0	0.0	115	39.0
	all	96	100.0	70	100.0	51	100.0	73	100.0	3	100.0	2	100.0	295	100.0
Deletion 13q	no	29	29.9	34	47.2	27	52.9	31	41.9	1	33.3	1	50.0	123	41.1
	yes	68	70.1	38	52.8	24	47.1	43	58.1	2	66.7	1	50.0	176	58.9
	all	97	100.0	72	100.0	51	100.0	74	100.0	3	100.0	2	100.0	299	100.0
Deletion 11q	no	79	81.4	64	90.1	41	80.4	56	75.7	0	0.0	2	100.0	242	81.2
	yes	18	18.6	7	9.9	10	19.6	18	24.3	3	100.0	0	0.0	56	18.8
	all	97	100.0	71	100.0	51	100.0	74	100.0	3	100.0	2	100.0	298	100.0
Deletion 17p	no	88	91.7	65	91.5	47	92.2	64	86.5	3	100.0	2	100.0	269	90.6
	yes	8	8.3	6	8.4	4	7.8	10	13.5	0	0.0	0	0.0	28	9.4
	all	96	100.0	71	100.0	51	100.0	74	100.0	3	100.0	2	100.0	297	100.0
Trisomy 12	no	87	89.7	57	80.3	40	78.4	66	89.2	3	100.0	2	100.0	255	85.6
	yes	10	10.3	14	19.7	11	21.6	8	10.8	0	0.0	0	0.0	43	14.4
	all	97	100.0	71	100.0	51	100.0	74	100.0	3	100.0	2	100.0	298	100.0

Supplementary Table 10)

Multivariable Cox proportional hazards regression analysis in CLL and REACH for OS and PFS.

CLL8

	HR	95% CI	*p*-value
Treatment FCR	0.67	[0.46, 0.97]	0.04
Cluster EMT-L	1.13	[0.56, 2.29]	0.74
Cluster (I)GI	1.52	[0.86, 2.68]	0.15
Cluster GI	1.35	[0.84, 2.14]	0.21
TP53 mutation	2.22	[1.25, 3.95]	0.007
IGHV unmutated	2.07	[1.22, 3.52]	0.007
del(13q) single	0.92	[0.54, 1.57]	0.76
del(11q)	1.12	[0.71, 1.77]	0.64
del(17p)	3.23	[1.68, 6.23]	< 0.001
tri(12)	1.15	[0.58, 2.26]	0.68

OS: Cox regression model, *n* = 307, number of events = 116.

	HR	95% CI	*p*-value
Treatment FCR	0.51	[0.38, 0.68]	< 0.001
Cluster EMT-L	0.97	[0.57, 1.66]	0.92
Cluster (I)GI	1.17	[0.78, 1.77]	0.45
Cluster GI	1.29	[0.93, 1.80]	0.13
TP53 mutation	1.82	[1.06, 3.13]	0.03
IGHV unmutated	1.59	[1.12, 2.25]	0.01
del(13q) single	1.04	[0.71, 1.54]	0.83
del(11q)	1.83	[1.27, 2.63]	0.001
del(17p)	4.78	[2.42, 9.45]	< 0.001
tri(12)	0.92	[0.55, 1.53]	0.74

PFS: Cox regression model, *n* = 307, number of events = 217.

REACH

	HR	95% CI	*p*-value
Treatment FCR	0.91	[0.65, 1.28]	0.59
Cluster EMT-L	1.04	[0.64, 1.69]	0.87
Cluster (I)GI	1.07	[0.64, 1.79]	0.80
Cluster GI	1.13	[0.72, 1.76]	0.60
TP53 mutation	1.39	[0.73, 2.65]	0.31
IGVH unmutated	2.43	[1.60, 3.70]	< 0.001
del(13q) single	1.20	[0.82, 1.76]	0.35
del(11q)	1.14	[0.73, 1.78]	0.56
del(17p)	2.66	[1.38, 5.12]	0.004
tri(12)	0.79	[0.46, 1.34]	0.38

OS: Cox regression model, *n* = 266, number of events = 140.

	HR	95% CI	*p*-value
Treatment FCR	0.68	[0.51, 0.89]	0.006
Cluster EMT-L	1.35	[0.92, 1.99]	0.13
Cluster (I)GI	1.21	[0.80, 1.83]	0.36
Cluster GI	1.49	[1.03, 2.15]	0.04
TP53 mutation	1.23	[0.72, 2.10]	0.44
IGVH unmutated	1.84	[1.34, 2.54]	< 0.001
del(13q) single	1.15	[0.84, 1.58]	0.37
del(11q)	1.11	[0.76, 1.61]	0.60
del(17p)	1.94	[1.11, 3.39]	0.02
tri(12)	1.19	[0.78, 1.82]	0.43

PFS: Cox regression model, *n* = 266, number of events = 212.
