# Supplementary material for: Multi-platform profiling characterizes molecular subgroups and resistance networks in chronic lymphocytic leukemia
Source: Nat Commun. 2021 Sep 13;12:5395. doi: 10.1038/s41467-021-25403-y (PMC8438057; doi:10.1038/s41467-021-25403-y)
Supplement: Supplementary file 3 — Reporting summary [file 41467_2021_25403_MOESM3_ESM.pdf]

nature research

Corresponding author(s): Johannes Bloehdorn

Last updated by author(s): 18.06.2021

Reporting Summary

Nature Research wishes to improve the reproducibility of the work that we publish. This form provides structure for consistency and transparency in reporting. For further information on Nature Research policies, see Authors & Referees and the Editorial Policy Checklist .

Statistics

For all statistical analyses, confirm that the following items are present in the figure legend, table legend, main text, or Methods section.

n/a	Confirmed	
☐	☒	The exact sample size (*n*) for each experimental group/condition, given as a discrete number and unit of measurement
☐	☒	A statement on whether measurements were taken from distinct samples or whether the same sample was measured repeatedly
☐	☒	The statistical test(s) used AND whether they are one- or two-sided<br>*Only common tests should be described solely by name; describe more complex techniques in the Methods section.*
☐	☒	A description of all covariates tested
☐	☒	A description of any assumptions or corrections, such as tests of normality and adjustment for multiple comparisons
☐	☒	A full description of the statistical parameters including central tendency (e.g. means) or other basic estimates (e.g. regression coefficient) AND variation (e.g. standard deviation) or associated estimates of uncertainty (e.g. confidence intervals)
☐	☒	For null hypothesis testing, the test statistic (e.g. *F*, *t*, *r*) with confidence intervals, effect sizes, degrees of freedom and *P* value noted<br>*Give P values as exact values whenever suitable.*
☒	☐	For Bayesian analysis, information on the choice of priors and Markov chain Monte Carlo settings
☒	☐	For hierarchical and complex designs, identification of the appropriate level for tests and full reporting of outcomes
☐	☒	Estimates of effect sizes (e.g. Cohen's *d*, Pearson's *r*), indicating how they were calculated

Our web collection on statistics for biologists contains articles on many of the points above.

Software and code

Policy information about availability of computer code

Data collection	Not used
Data analysis	RNA quantification and quality control was performed with the Agilent 2100 Bioanalyzer and 2100 Expert Software Version 2.6 (B.02.07.SI532). Affymetrix Human Exon 1.0 ST Array (HuEx-1_0-st-v2) data files were preprocessed by the robust multichip average (RMA) algorithm using the aroma.affymetrix R package version 2.12.0 (Bengtsson H et al. Methods. 2008;Tech Repor(745):1-9 2008). Statistical procedures on expression data were performed with the R software environment, versions 3.3.3 and 3.4.1 and BRB-ArrayTools Versions 4.2.1 - 4.6.1 (available at http://linus.nci.nih.gov/BRB-ArrayTools.html and www.r-project.org). Agglomerative hierarchical clustering and consensus clustering (Monti S et. al. Mach Learn. 2003;52:91-118) was applied (R environment or Genesis, release 1.8.0). Visualizing selected gene sets was conducted using the Genesis platform, release 1.8.0 (Sturn A et al. Bioinformatics. 2002;18 (1):207-208). Analysis on dominant biological processes was performed with the Gene Set Enrichment Analysis (GSEA) (Subramanian A et. al. Proc Natl Acad Sci U S A. 2005;102(43):15545-15550), release v3.0 . SNP genotype calls were generated by applying the birdseed algorithm in Genotyping Console version 4.0 (Affymetrix) using at least 50 arrays in each analysis. DNA copy number analyses were performed using reference alignment (Pounds S et. al. Bioinformatics. 2009;25(3):315-321), dChip version 2010.01 (dChipSNP: Lin M et.al. Bioinformatics. 2004;20(8):1233-1240) and circular binary segmentation (CBS) (Olshen AB. Biostatistics. 2004;5(4):557-572), DNACopy version 1.44.0. Aroma.affymetrix software package (Bengtsson H et.al. Bioinformatics. 2008;24(6):759-767) was used for an exact determination of segment boundaries. Size position and location of genes were identified with the UCSC Genome Browser; assembly March 2006, NCBI36/hg18 (http://www.genome.ucsc.edu/) (Kent W et. al. UCSC Genome Browser. Hum genome Brows UCSC Genome Res. 2002). For visualization of deletion size for del(13q) the Integrative Genomics Viewer, release 2.4.16, was used (Robinson JT et al. Nat Biotechnol. 2011). To assess the specific enrichment of genomic amplifications and deletions within clusters identified by consensus clustering of GEP, we applied GISTIC (Beroukhim R et. al. Proc Natl Acad Sci. 2007;104(50):20007-20012), release v2.0.23, to the curated SNP array dataset. We used non-negative matrix factorization to assess the pathogenic processes operational in identified CLL subtypes which best explain the mutation pattern observed in corresponding cases (Alexandrov LB et al. Nature. 2013;500 (7463):415-421; Seung HS et al. Nature 1999;401(6755):788-791). Related statistics were performed with MATLAB 2018b. Signature projections for mutational processes were conducted and analyzed by using the SignatureAnalyzer algorithm (https:// software.broadinstitute.org/cancer/cga/msp). Intensities of individual bands in western blots were analyzed using Fiji ImageJ

densitometry software, version 1.51j. Statistical analysis was further performed with software environment R, versions 3.3.3 and 3.4.1 with the R package survival, version 2.41-2; SPSS version 24-26 (IBM, NYC, NY) and Prism software version 6.0h (GraphPad). Images (fluorescence) were taken using Nikon Ci-L upright fluorescence microscope and Nikon NIS Elements AR software (Ver4. 30.01, 64bit edition). For comet assay image analysis an open source Cell Profiler (https://cellprofiler.org/) software equipped with the Comet Assay analysis module (https://cellprofiler-examples.s3.amazonaws.com/ExampleCometAssay.zip) was used. Proteomics raw data was analyzed by Proteome Discoverer 1.4.1.14 with SequestHT 1.1.1.11 and Percolator modules searched against the mouse UniProt Swissprot and trembl databases (downloaded 01/15). For reduced representation bisulfite sequencing libraries were sequenced and aligned to the bisulfite-converted hg19 reference genome using Bismark (RRID: SCR_005604) v0.15.0 (Krueger, F. & Andrews, S. R. Bismark: A flexible aligner and methylation caller for Bisulfite-Seq applications. Bioinformatics 2011 Jun 1;27(11) 1571–1572).

For manuscripts utilizing custom algorithms or software that are central to the research but not yet described in published literature, software must be made available to editors/reviewers. We strongly encourage code deposition in a community repository (e.g. GitHub). See the Nature Research guidelines for submitting code & software for further information.

Data

Policy information about availability of data

All manuscripts must include a data availability statement. This statement should provide the following information, where applicable:

- Accession codes, unique identifiers, or web links for publicly available datasets
- A list of figures that have associated raw data
- A description of any restrictions on data availability

Complete data sets are available: For GEP at Gene Expression Omnibus (http://www.ncbi.nlm.nih.gov/geo/; GEO accession number: GSE58211 (REACH only); GSE126595 (full clinical data set); GSE126699 (including functional data). For SNP-Microarray raw data at Gene Expression Omnibus (GEO accession number: GSE36908 (CLL8 treatment naive) and GSE83566 (relapsed)). CLL8 WES data is deposited in dbGaP under accession code phs000922.v1.p1. CLL8 RRBS sequencing data is available from the NCBI (GEO accession number: GSE143673). The proteome profiling raw data and processed outputs are available at https://www.ebi.ac.uk/pride/archive/projects/PXD004608.

Field-specific reporting

Please select the one below that is the best fit for your research. If you are not sure, read the appropriate sections before making your selection.

- [x] Life sciences
- [ ] Behavioural & social sciences
- [ ] Ecological, evolutionary & environmental sciences

For a reference copy of the document with all sections, see nature.com/documents/nr-reporting-summary-flat.pdf

Life sciences study design

All studies must disclose on these points even when the disclosure is negative.

Sample size	Patient samples for multi-platform profiling were used based on availability of material with highest quality from the underlying clinical trial cohorts (CLL8 (GEP:n=426) and the REACH(GEP:n=300) trials). Sample size for the validation cohort closely matched the discovery cohort.
Data exclusions	Data was analysed as given, no data exclusion performed.
Replication	Gene expression profiles from independent patient samples of the randomized, multicenter phase 3 CLL8 trial were validated on expression profiles from independent patient samples of the randomized, multicenter phase 3 REACH trial. No replicates were used from individual samples for gene expression profiling. Confirmation of biological categories and processes inferred from the multi-platform analysis was conducted in multiple, independent in vitro and in vivo mouse models. Experimental findings were reliably reproduced. All experiments have been performed with appropriate replicates as described in the Figures and Methods sections.
Randomization	Patient samples were taken from 2 independent randomized trials. In vivo validation was conducted on defined mouse models and therefore no randomization was needed.
Blinding	Due to the experimental design and sequential analysis workflow sample blinding was not neccessary for multi-platform profiling and confirmatory mouse models. Visual quantifications were performed in a blinded manner as specified in the respective manuscript sections.

Reporting for specific materials, systems and methods

We require information from authors about some types of materials, experimental systems and methods used in many studies. Here, indicate whether each material, system or method listed is relevant to your study. If you are not sure if a list item applies to your research, read the appropriate section before selecting a response.

Materials & experimental systems

n/a	Involved in the study
☐	☒ Antibodies
☐	☒ Eukaryotic cell lines
☒	☐ Palaeontology
☐	☒ Animals and other organisms
☐	☒ Human research participants
☐	☒ Clinical data

Methods

n/a	Involved in the study
☒	☐ ChIP-seq
☒	☐ Flow cytometry
☒	☐ MRI-based neuroimaging

Antibodies

Antibodies used

Antibody: anti-AKT, Product number:#9272, Company: Cell Signaling. Antibody: anti-c-Myc, Product number:#ab32072, Company: Abcam. Antibody: anti-ERK1 (k-23), Product number:#sc-94, Company: Santa Cruz. Antibody: anti-p53(CM5), Product number:#554293, Company: BD Bioscience. Antibody: anti-phospho-AKT (Thr308), Product number:#4056, Company: Cell Signaling. Antibody: anti-phospho-ERK(E-4), Product number:#sc-7383, Company: Santa Cruz. Antibody: anti-phospho-p53 (ser15), Product number:#9286, Company: Cell Signaling. Antibody: anti-PRMT5, Product number:#2252, Company: Cell Signaling. Antibody: anti-RB(C-15), Product number:#sc-50, Company: Santa Cruz. Antibody: anti-XPO1/CRM1 (H-300), Product number:#sc-5595, Company: Santa Cruz. Antibody: anti-ß-Actin, Product number:#sc-1615, Company: Santa Cruz. Antibody: anti-yH2AX, Product number: ab26350, Company: Abcam. Antibody: anti-mouse Alexa Fluor 594, Product number: ab150116, Company: Abcam. Antibody: HRP-conjugated anti-mouse, Product number: A16072, Company: Thermo Fisher. Antibody: anti-GAPDH, Product number: ab8245, Company: Abcam.

Validation

All antibodies used in this study were validated by the manufacturers for specific detection of the antigen and species reactivity. Specifications and validation data is available on the homepage of the manufacturer. Exemplary references are listed below for used antibodies and applications, multiple additional references are available from the manufacturer homepage.

p53(CM5): Nucleoporin Nup155 is part of the p53 network in liver cancer. Nature Communications 14 May 2019 by Holzer, K., Ori, A., et al.. Species: Human, Application: WB
Phospho-p53 (ser15): A Compendium of Mutational Signatures of Environmental Agents. Cell, 2 May 2019 by Kucab, J. E., Zou, X., et al.. Species: Human, Application: WB
PRMT5: Coordinated Splicing of Regulatory Detained Introns within Oncogenic Transcripts Creates an Exploitable Vulnerability in Malignant Glioma. Cancer Cell, 9 October 2017 by Braun, C. J., Stanciu, M., et al.. Species: Human, Application: WB
XPO1/CRM1 (H-300): XPO1-dependent nuclear export is a druggable vulnerability in KRAS-mutant lung cancer. Nature, 6 October 2016 by Kim, J., McMillan, E., et al.. Species: Human, Application: WB
c-Myc: A living biobank of ovarian cancer ex vivo models reveals profound mitotic heterogeneity. Nature Communications, 13 February 2020 by Nelson, L., Tighe, A., et al.. Species: Human, Application: WB
Actin: The K219T-Lamin mutation induces conduction defects through epigenetic inhibition of SCN5A in human cardiac laminopathy. Nature Communications, 22 May 2019 by Salvarani, N., Crasto, S., et al..Species: Human, Application: WB
AKT / Phospho-AKT (Thr308): Phenotypic profiling with a living biobank of primary rhabdomyosarcoma unravels disease heterogeneity and AKT sensitivity. Nat Commun 2020 Sep 15 Gabriele Manzella, et. al. Species: Human, Application: WB
ERK1: Anti-invasive effects of CXCR4 and FAK inhibitors in non-small cell lung carcinomas with mutually inactivated p53 and PTEN tumor suppressors. Dragoj, M. et al. 2017. Investigational new drugs. 35: 718-732. Species: Human, Application: WB
Phospho-ERK: Pseudomonas aeruginosa induces p38MAP kinase-dependent IL-6 and CXCL8 release from bronchial epithelial cells via a Syk kinase pathway. Coates, MS.|Alton, EWFW.|Rapeport, GW.|Davies, JC.|Ito, K.| et al. 2021. PLoS One. 16: e0246050. Species: Human, Application: WB
RB: Haploinsufficiency for BRCA1 leads to cell-type-specific genomic instability and premature senescence. Sedic, M. et al. 2015. Nat Commun. 6: 7505. Species: Human, Application: WB
anti-yH2AX: N6-methyladenosine regulates glycolysis of cancer cells through PDK4. Li Z et al. Nat Commun 2020 11:2578. Species: Human, Application: WB
Alexa Fluor 594: Interferon inducible X-linked gene CXorf21 may contribute to sexual dimorphism in Systemic Lupus Erythematosus. Odhams CA et al. Nat Commun 2019 10:2164. Species: Human, Application: WB
HRP-conjugated anti-mouse: Effects of Hylomecon vernalis ethanol extracts on cell cycle and apoptosis of colon cancer cells Jing Sun et al. Mol Med Rep 2017 Jun;15(6):3485-3492. Species: Human, Application: WB
GAPDH: Leucine regulates autophagy via acetylation of the mTORC1 component raptor. Son SM et al. Nat Commun 2020 11:3148. Species: Human, Application: WB

Eukaryotic cell lines

Policy information about cell lines

Cell line source(s)	Cell lines EHEB, GRANTA, JVM2, JVM3, MEC1, MEC2, LCL-WEI and BL2 were purchased from the German Collection of Microorganisms and Cell culture. Human embryonic kidney HEK293T were obtained from European Collection of Authenticated Cell Cultures (ECACC) (Culture Collections, Public Health England, Salisbury, UK). BL2 AICDA- were kindly provided by Claude-Agnes Reynaud (INSERM U1151, Paris) and were described previously (Faili A, Aoufouchi S, Guéranger Q, et al. AID-dependent somatic hypermutation occurs as a DNA single-strand event in the BL2 cell line. Nat Immunol. 2002 Sep;3(9):815-21).
Authentication	Cell lines EHEB, GRANTA, JVM2, JVM3, MEC1, MEC2, LCL-WEI, BL2 were purchased with the certificate from the vendor: German Collection of Microorganisms and Cell culture (Deutsche Sammlung von Mikroorganismen und Zellkulturen, DSMZ) and EHEB, GRANTA, JVM2, JVM3, MEC1, MEC2, LCL-WEI were additionally authenticated through sequencing by Multiplexion GmbH. Human embryonic kidney HEK293T were obtained from European Collection of Authenticated Cell Cultures (ECACC) (Culture Collections, Public Health England, Salisbury, UK). BL2 AICDA- cells were not authenticated in-house.
Mycoplasma contamination	All cells were tested for mycoplasma contamination monthly.
Commonly misidentified lines (See ICLAC register)	None

Animals and other organisms

Policy information about studies involving animals; ARRIVE guidelines recommended for reporting animal research

Laboratory animals	8-12 week old female WT Balb/c and C57BL6/J mice; Eμ-myc [C57BL/6J-TgN(Ighmyc)22Bri/J] hemizygous, Eμ-TCL1 [C57BL/6J-TgN (IghTCL1)22Bri/J] hemizygous mice and wildtype mice aged 6 weeks and 200 days all female.
Wild animals	No wild animals were used in this study.
Field-collected samples	No field-collected samples were used in this study.
Ethics oversight	TCL1 serial transplant mouse model was performed according to protocols approved by the state government of Baden-Wuerttemberg, following the animal welfare guidelines (Registration 1124 and 1128) and were approved by the Ulm University animal experimental ethics committee. BCL1 syngeneic transplant model, Eμ-Myc / Eμ-TCL1 mouse model were conducted under the Home Office licenses PPL30/2964 and P4D9C89EA following approval by local ethical committees, reporting to the Home Office Animal Welfare Ethical Review Board (AWERB) at the University of Southampton.

Note that full information on the approval of the study protocol must also be provided in the manuscript.

Human research participants

Policy information about studies involving human research participants

Population characteristics	The cohort analysed in this study contained peripheral blood samples from participants enrolled onto the CLL8 trial between 2003-2007 (median age 61 years, 74.3% men) and GEP/genetics data from patients samples enrolled onto the REACH trial between 2003-2008 (median age 62 years, 66.7% men). Samples were taken at enrollment onto the respective trials before initiation of treatment (CLL8 n=426; REACH n=300). Patient characteristics (e.g. age, genetics, biological markers) were representative for first-line and second-line treatment conditions as compared to the general population of CLL patients with need for treatment and compared to the whole trial populations.
Recruitment	Samples used in this study were chosen based on availability and sufficient quality of the material. Higher leukocyte counts were observed for the CLL8 discovery cohort of CD19 sorted CLL cases, likely through selection of samples with abundant material for multiple analyses, however, patient characteristics and especially high-risk markers showed a well-balanced distribution representative of the full trial population. Assessment of batch effects imposed through e.g. time point of sampling, location of sampling, time point of labeling/hybridization and other factors could not be observed. Only samples from patients with need for treatment and fulfilling inclusion/exclusion crieria, as defined in the respective study protocol, were collected at enrolment onto the respective trials at participating centers.
Ethics oversight	Trial participation, genetic testing and data evaluation have been conducted after informed patient consent, with the approval of the respective local ethics committees of participating centers and in accordance with the Declaration of Helsinki. Data analysis and conductance of multi-platform profiling in this study was approved by the Ulm University ethics committee.

Note that full information on the approval of the study protocol must also be provided in the manuscript.

Clinical data

Policy information about clinical studies

All manuscripts should comply with the ICMJEguidelines for publication of clinical research and a completedCONSORT checklist must be included with all submissions.

Clinical trial registration	The clinical trials are registered on Clinicaltrials.gov under NCT00281918 and NCT00090051.
Study protocol	Further information is given at Clinicaltrials.gov and the original publications for CLL8 (Hallek M. et al. Lancet. 2010 Oct 2;376 (9747):1164-74) and REACH(Robak T et. al. J Clin Oncol. 2010;28(10):1756-1765).
Data collection	Both interventional trials were designed as international, open-label, multicenter, randomized, comparative, Phase III trials being conducted between 2003-2007 (CLL8) and 2003-2008 (REACH). Survival data was collected for a median observation time of 5.9 (CLL8) and 4.9 (REACH) years. Data for the CLL8 trial was collected centrally at the trial office of the German CLL Study Group in Cologne.
Outcomes	Details are described in the original publications for CLL8 (Hallek M. et al. Lancet. 2010 Oct 2;376(9747):1164-74) and RACH (Robak T et. al. J Clin Oncol. 2010;28(10):1756-1765).
